# Mononuclear Tricoordinate Copper(I) and Silver(I) Halide Complexes of a Sterically Bulky Thiourea Ligand and a Computational Insight of Their Interaction with Human Insulin

**DOI:** 10.3390/molecules27134231

**Published:** 2022-06-30

**Authors:** Awal Noor, Sadaf Qayyum, Farukh Jabeen, Ashfaq Ur Rehman

**Affiliations:** 1 Department of Basic Sciences, Preparatory Year Deanship, King Faisal University, Al-Hassa 31982, Saudi Arabia; sqayyum@kfu.edu.sa; 2 Department of Chemistry and Biochemistry, Laurentian University, 935 Ramsey Lake Road, Sudbury, ON P3E 2C6, Canada; fjabeen@laurentian.ca; 3 Department of Molecular Biology and Biochemistry, University of California, Irvine, CA 92697-3900, USA; aurehman@uci.edu

**Keywords:** copper, coordination chemistry, human insulin, metal complexes, molecular docking, silver, S-ligands, thiourea, X-ray structures

## Abstract

Reaction of two equivalents of the bulky 1,3-bis(2,6-diethylphenyl)thiourea ligand (**L**) with MX (being M = Cu^+^, Ag+; and X = Cl^−^, Br^−^, I^−^) in acetonitrile afforded neutral complexes of the type [MXL_2_] [CuClL_2_].2CH_3_CN (**1a**); [CuBrL_2_].2CH_3_CN (**1b**); [CuIL_2_] (**1c**): [AgClL_2_] (**2a**); [AgBrL_2_] (**2b**) and [AgIL_2_] (**2c**). The two aromatic groups in free ligand were found to be *trans* with respect to the thiourea unit, which was a reason to link the ligand molecules via intermolecular hydrogen bonding. Intramolecular hydrogen bonding was observed in all metal complexes. The copper complexes **1a** and **1b** are acetonitrile solvated and show not only intra- but also intermolecular hydrogen bonding between the coordinated thiourea and the solvated acetonitrile molecules. Silver complexes reported here are the first examples of structurally characterized tricoordinated thiourea-stabilized monomeric silver(I) halides. Molecular docking studies were carried out to analyze the binding modes of the metal complexes inside the active site of the human insulin (HI) protein. Analysis of the docked conformations revealed that the electrostatic and aromatic interactions of the protein *N*-terminal residues (i.e., Phe and His) may assist in anchoring and stabilizing the metal complexes inside the active site. According to the results of docking studies, the silver complexes exhibited the strongest inhibitory capability against the HI protein, which possesses a deactivating group, directly bonded to silver. All compounds were fully characterized by elemental analysis, NMR spectroscopy, and molecular structures of the ligand, and five out of six metal complexes were also confirmed by single-crystal X-ray diffraction.

## 1. Introduction

Thiourea ligands are of interest mainly due to their ready accessibility and broad applications in catalysis, biological activity, and optical technology [1,2,3,4]. The presence of soft sulfur and harder nitrogen donor atoms makes these ligands show different bonding possibilities and thus exhibit versatility in coordination chemistry. Thioureas can act as neutral, monoanionic, or dianionic ligands [5,6,7]. Despite the fact that it is a mature field of research, it nevertheless continues to attract the attention of synthetic chemists due to the possible variation of steric and electronic effects at the nitrogen atoms. This, in turn, alters their physical as well as chemical properties and has thus led to the isolation of not only efficient but also air- and moisture-stable catalysts, for instance for azide-alkyne addition and Heck reactions [8,9,10]. One of the focuses in the past has been the synthesis of *N*-alkyl/aryl- and *N*,*N*′-dialkyl/diaryl-substituted thioureas and their coordination chemistry [11,12,13,14,15,16,17]. Among structurally characterized tricoordinate univalent metals, copper has been extensively studied, and silver rarely studied [18,19,20]. In particular, anhydrous metal (I) halide complexes of symmetrical *N*,*N*′-diaryl-substituted thioureas are rare, and among them, the structurally characterized tricoordinate silver (I) halides of thiourea are unknown [8]. Thus, we were interested in synthesizing and structurally characterizing *N*,*N*′-diaryl-substituted thiourea metal complexes of univalent copper and silver. First reported in 1997, 1,3-bis(2,6-diethylphenyl)thiourea is a symmetrically substituted ligand [21]. Surprisingly, to the best of our knowledge, metal complexes of it have not been reported to date. In addition to their structural importance, thiourea derivatives are also known for their diverse biological applications. The thiourea complexes of Cu and Ag have received special attention in bioinorganic applications mainly due to their low toxicity and their binding ability to human proteins [22,23,24]. The ability of thiourea to form hydrogen bonds favorably affects the solubility of its complexes in an aqueous medium, which contributes to better penetration into cells [25]. In this context, biological applications of coordination compounds are of particular importance to us [26,27,28,29]. Thiourea derivatives and their transition metal complexes have been shown to be quite potent antidiabetic agents [30,31,32].

Molecular docking has emerged as one of the most efficient virtual screening methods. It offers the possibility to predict the binding affinity between the applied compound and the target protein, but it also provides a helpful insight into the structure of the protein–target complex. The approach has been the focus of recent research activities for the discovery of a great number of new drugs, despite the fact that it is still far from full success in terms of its success rate. The promising antidiabetic behavior of free thioureas and our recent contribution prompted us to investigate the potential of these thiourea complexes with the human insulin receptor sites using molecular docking studies [33]. Herein, we report the synthesis and detailed characterization of thiourea ligands stabilized with univalent copper and silver complexes and provide a theoretical insight into their interaction with human insulin.

## 2. Experimental Section

### 2.1. Materials and Methods

Analytical grade solvents were used without any further purification. Deuterated solvents were obtained from Cambridge Isotope Laboratories and were degassed and dried prior to use. Thiourea ligand (1,3-bis(2,6-diethylphenyl)thiourea = **L**) was obtained from the reaction of CS_2_ with a solution of 2,6-diethylaniline, trimethylamine, and water. Although the used ligand has been previously reported [21], we instead followed the synthetic methodology adopted by Cowley and co-workers [34]. NMR spectra were recorded on a Varian spectrometer at 300 MHz and 400 MHz at ambient temperature. The chemical shifts are reported in ppm relative to the internal TMS. Elemental analyses (CHN) were determined using a Vario EL III instrument. X-ray crystal structure analyses were performed by using a STOE-IPDS II and a STOE STADIVARI (λ(Mo-Kα) = 0.71073 Å) diffractometers equipped with Oxford Cryostream low-temperature units. Structure solution and refinement was accomplished using SIR97 [35], SHELXL2014 [36], WinGX [37], and Olex2 [38]. For **L** and **2c**, we observed one B-alert each in the checkcif files due to the. missing of low-angle reflections as a result of beam stop and bad crystal quality, respectively. Selected crystallographic data are gathered in Table 1.

### 2.2. Synthesis of Compounds ***1a**–**c*** and ***2a**–**c***

For the synthesis of **1a**, acetonitrile (30 mL) was added to ligand (0.169 g, 0.5 mmol) and CuCl (0.025 g, 0.25 mmol) at room temperature. The resulting suspension was stirred overnight, resulting in a small amount of white precipitation. The precipitate was separated by filtration, and the filtrate was allowed to afford colorless crystals of **1a**. Both precipitate and the crystalline material were identified to be the same material of the desired compound **1a**. Following the same procedure, **1b** was prepared by treating ligand (0.169 g, 0.5 mmol) with CuBr (0.36 g, 0.25 mmol). For **1c**, ligand (0.169 g, 0.5 mmol) was treated with CuI (0.048 g, 0.25 mmol). Acetonitrile was evaporated and the crystalline material was re-dissolved in CHCl_3_ and filtered. The filtrate was allowed to slowly evaporate to afford colorless crystals of **1c** at room temperature. Following the synthetic procedure of **1a**, compund **2a** was prepared by reacting ligand (0.169 g, 0.5 mmol) with AgCl (0.036 g, 0.25 mmol). For **2b**, ligand (0.228 g, 0.67 mmol) was reacted with AgBr (0.063 g, 0.33 mmol). Compound 2c was prepared by reacting ligand (0.169 g, 0.5 mmol) with AgI (0.059 g, 0.25 mmol). Acetonitrile was evaporated, and the crystalline material was re-dissolved in CHCl_3_ and filtered. Slow evaporation of solvent from filtrate at room temperature afforded colorless crystals of **2c**.

**Complex 1a:** Yield: 90% (0.175 g). EA: Calculated for C_42_H_56_CuClN_4_S_2_ (780.05): C 64.67, H 7.24, N 7.18; found: C 64.80, H 7.00, N 7.14. ^1^H NMR (CDCl_3_, 400 MHz): *δ* = 1.13 (t, 12H, H*^CH3^*^(CH2)^, *J* = 7.4 Hz), 1.35 (t, 12H, H*^CH3^*^(CH2)^, *J* = 7.4 Hz), 2.55–2.70 (m, 16H, H^CH3(*CH2*))^, 2.92–3.00 (m, 4H, H^CH3(*CH2*))^, 6.35 (s, 2H, H^NH^), 7.09 (d, 8H, H*^C6H5^*, *J* = 7.4 Hz), 7.23 (d, 8H, H*^C6H5^*, *J* = 7.4 Hz), 7.34 (t, 4H, H*^C6H5^*, *J* = 7.4 Hz), 10.52 (s, 2H, H*^NH^*) ppm. ^13^C NMR (CDCl_3_, 100 MHz): *δ* = 14.5 (C*^CH3^*^(CH2)^), 14.7 (C*^CH3^*^(CH2)^), 24.5 (C^CH3(*CH2*)^), 24.8 (C^CH3(*CH2*)^), 126.4 (C*^CH^*), 127.0 (C*^CH^*), 128.8 (C*^CH^*), 129.6 (C*^CH^*), 131.9 (C*^C^*), 133.3 (C*^C^*), 142.0 (C*^C^*), 143.0 (C*^C^*), 178.9 (C*^C=S^*) ppm.

**Complex 1b:** Yield: 74% (0.210 g). EA: Calculated for C_42_H_56_CuBrN_4_S_2_ (824.50): C 61.18, H 6.85, N 6.90; found: C 61.22, H 7.10, N 6.99. ^1^H NMR (CDCl_3_, 300 MHz): *δ* = 1.14 (t, 12H, H*^CH3^*^(CH2)^, *J* = 7.5 Hz), 1.38 (t, 12H, H*^CH3^*^(CH2)^, *J* = 7.5 Hz), 2.58 (m, 8H, H^CH3(*CH2*)^), 2.64 (q, 4H, H^CH3(*CH2*)^), 2.98 (m, 4H, H^CH3(*CH2*)^), 6.35 (s, 2H, H*^NH^*), 7.07–7.40 (m, 12H, H*^C6H3^*), 10.28 (s, 2H, H*^NH^*) ppm. ^13^C NMR (CDCl_3_, 75 MHz): *δ* = 14.4 (C*^CH3^*^(CH2^)), 14.8 (C*^CH3^*^(CH2)^), 24.4 (C^CH3(*CH2*)^), 24.7 (C^CH3(*CH2*)^), 126.2 (C*^CH^*), 126.9 (C*^CH^*), 128.7 (C*^CH^*), 129.7 (C*^CH^*), 131.7 (C*^C^*), 133.1 (C*^C^*), 141.9 (C*^C^*), 143.0 (C*^C^*), 178.6 (C*^C=S^*) ppm.

**Complex 1c:** Yield: 93% (0.200 g). EA: Calculated for C_42_H_56_CuIN_4_S_2_ (871.50): C 57.88, H 6.48, N 6.43; found: C 57.59, H 6.38, N 6.37. ^1^H NMR (CDCl_3_, 300 MHz): *δ* = 1.13 (t, 12H, H*^CH3^*^(CH2)^, *J* = 7.5 Hz), 1.38 (t, 12H, H*^CH3^*^(CH2)^, *J* = 7.5 Hz), 2.58 (q, 8H, H^CH3(*CH2*)^, *J* = 7.5 Hz), 2.70 (q, 4H, H^CH3(*CH2*)^, *J* = 7.5 Hz), 2.97 (q, 4H, H^CH3(*CH2*)^, *J* = 7.5 Hz), 6.37 (s, 2H, H*^NH^*), 7.06–7.40 (m, 12H, H*^C6H3^*), 9.98 (s, 2H, H*^NH^*) ppm. ^13^C NMR (CDCl_3_, 75 MHz): *δ* = 14.3 (C*^CH3^*^(CH2)^), 15.0 (C*^CH3^*^(CH2)^), 24.3 (C^CH3(*CH2*)^), 24.3 (C^CH3(*CH2*)^), 126.1 (C*^CH^*), 126.9 (C*^CH^*), 128.6 (C*^CH^*), 129.7 (C*^CH^*), 131.3 (C*^C^*), 132.9 (C*^C^*), 141.8 (C*^C^*), 143.0 (C*^C^*), 178.0 (C*^C=S^*) ppm.

**Complex 2a:** Yield: 91% (0.188 g). EA: Calculated for C_42_H_56_AgClN_4_S_2_ (824.37): C 61.19, H 6.85, N 6.80; found: C 60.57, H 6.90, N 6.70. ^1^H NMR (CDCl_3_, 300 MHz): *δ* = 1.17 (t, 12H, H*^CH3^*^(CH2)^, *J* = 7.75 Hz), 1.38 (t, 12H, H*^CH3^*^(CH2)^, *J* = 7.75 Hz), 2.57 (q, 8H, H^CH3(*CH2*)^, *J* = 7.75 Hz), 2.68–2.76 (m, 4H, H^CH3(*CH2*)^), 2.94–3.06 (m, 4H, H^CH3(*CH2*)^), 6.45 (s, 2H, H*^NH^*), 7.12 (d, 8H, H*^C6H5^*, *J* = 7.2 Hz), 7.25 (d, 8H, H*^C6H5^*, *J* = 7.75 Hz), 7.31–7.34 (m, 4H, H*^C6H5^*), 10.86 (s, 2H, H*^NH^*) ppm. ^13^C NMR (CDCl_3_, 75 MHz): *δ* = 14.4 (C*^CH3^*^(CH2)^), 14.5 (C*^CH3^*^(CH2)^), 24.4 (C^CH3(*CH2*)^), 24.7 (C^CH3(*CH2*)^), 126.3 (C*^CH^*), 126.8 (C*^CH^*), 128.7 (C*^CH^*), 129.6 (C*^CH^*), 131.7 (C*^C^*), 133.4 (C*^C^*), 142.0 (C*^C^*), 142.9 (C*^C^*), 178.7 (C*^C=S^*) ppm.

**Complex 2b:** Yield: 92% (0.267 g). EA: Calculated for C_42_H_56_AgBrN_4_S_2_ (868.82): C 58.06, H 6.50, N 6.45; found: C 57.76, H 6.19, N 6.48. ^1^H NMR (CDCl_3_, 400 MHz): *δ* = 1.14 (t, 12H, H*^CH3^*^(CH2)^, *J* = 7.5 Hz), 1.36 (t, 12H, H*^CH3^*^(CH2)^, *J* = 7.5 Hz), 2.55–2.72 (m, 16H, H^CH3(*CH2*)^), 2.92–3.01 (m, 4H, H^CH3(*CH2*)^), 6.43 (s, 2H, H*^NH^*), 7.11 (d, 8H, H*^C6H5^*, *J* = 7.5 Hz), 7.25 (d, 8H, H*^C6H5^*, *J* = 7.5 Hz), 7.35 (t, 4H, H*^C6H5^*, *J* = 7.5 Hz), 10.0 (s, 2H, H*^NH^*) ppm. ^13^C NMR (CDCl_3_, 100 MHz): *δ* = 14.5 (C*^CH3^*^(CH2)^), 14.7 (C*^CH3^*^(CH2)^), 24.5 (C^CH3(*CH2*)^), 24.9 (C^CH3(*CH2*)^), 126.5 (C*^CH^*), 127.0 (C*^CH^*), 128.9 (C*^CH^*), 129.8 (C*^CH^*), 131.7 (C*^C^*), 133.5 (C*^C^*), 142.1 (C*^C^*), 143.1 (C*^C^*), 179.1 (C*^C=S^*) ppm.

**Complex 2c:** Yield: 94% (0.214 g). EA: Calculated for C_42_H_56_AgIN_4_S_2_ (915.83): C 55.08, H 6.16, and N 6.12; found: C 55.04, H 5.85, and N 6.19. ^1^H NMR (CDCl_3_, 300 MHz): *δ* = 1.14 (t, 12H, H*^CH3^*^(CH2)^, *J* = 7.5 Hz), 1.35 (t, 12H, H*^CH3^*^(CH2)^, *J* = 7.5 Hz), 2.60 (q, 8H, H^CH3(*CH2*)^, *J* = 7.5 Hz), 2.69 (m, 4H, H^CH3(*CH2*)^), 2.99 (m, 4H, H^CH3(*CH2*)^), 6.43 (s, 2H, H*^NH^*), 7.08–7.37 (m, 12H, H*^C6H3^*), 9.31 (s, 2H, H*^NH^*) ppm. ^13^C NMR (CDCl_3_, 75 MHz): *δ* = 14.4 (C*^CH3^*^(CH2)^), 14.9 (C*^CH3^*^(CH2)^), 24.4 (C^CH3(*CH2*)^), 24.7 (C^CH3(*CH2*)^), 126.2 (C*^CH^*), 126.9 (C*^CH^*), 128.5 (C*^CH^*), 129.7 (C*^CH^*), 131.5 (C*^C^*), 133.6 (C*^C^*), 141.9 (C*^C^*), 143.2 (C*^C^*), 179.7 (C*^C=S^*) ppm.

### 2.3. Molecular Docking

The synthesized metal-based ligand complexes were studied using a molecular docking simulation with the crystal structure of human insulin (HI) as a target protein. The HI was retrieved from the RCSB protein data bank (https://www.rcsb.org/ accessed on 27 February 2022). The co-crystallized macromolecule cucurbit [7] uril is included in the HI protein structure. In order to elucidate the molecular mechanism of inhibition, the metal complexes (**1a**–**c** and **2a**–**c**) were docked in the active sites of the HI protein using the molecular operating environment (MOE) v2020 tool [39] and PDB code 3Q6E [40]. The target was prepared using the protein preparing module of the MOE. All solvent molecules were removed before the docking studies could begin. The cleft-like active site was mapped around the cognate ligand. Highly stiff complexes were found to benefit from the HI protein’s availability of a cleft-like pocket. As an active site, we leveraged that cleft. Finally, refined crystal structure was used for further docking study using the default parameters of MOE (i.e., Placement: Triangle Matcher, Rescoring 1: London dG, Refinement: Forcefield, Rescoring 2: GBVI/WSA). For the ligand, a total of 50 conformations were chosen before executing the docking approach. For the protein–ligand interaction (PLI) study, the top-ranked conformations by docking score and binding energy were chosen. Additionally, we have evaluated the specificity of the target with the metal complex using dynamut and WEBnm [41,42].

## 3. Results and Discussion

Structures of substituted thioureas have always been of interest mainly due to the difficulty in obtaining crystals suitable for X-ray analysis [43,44,45,46,47,48,49,50,51,52,53,54,55,56]. Crystals of ligand **L** partially suitable for X-ray analysis were grown from its chloroform solution by slow evaporation.

**Figure 1:**
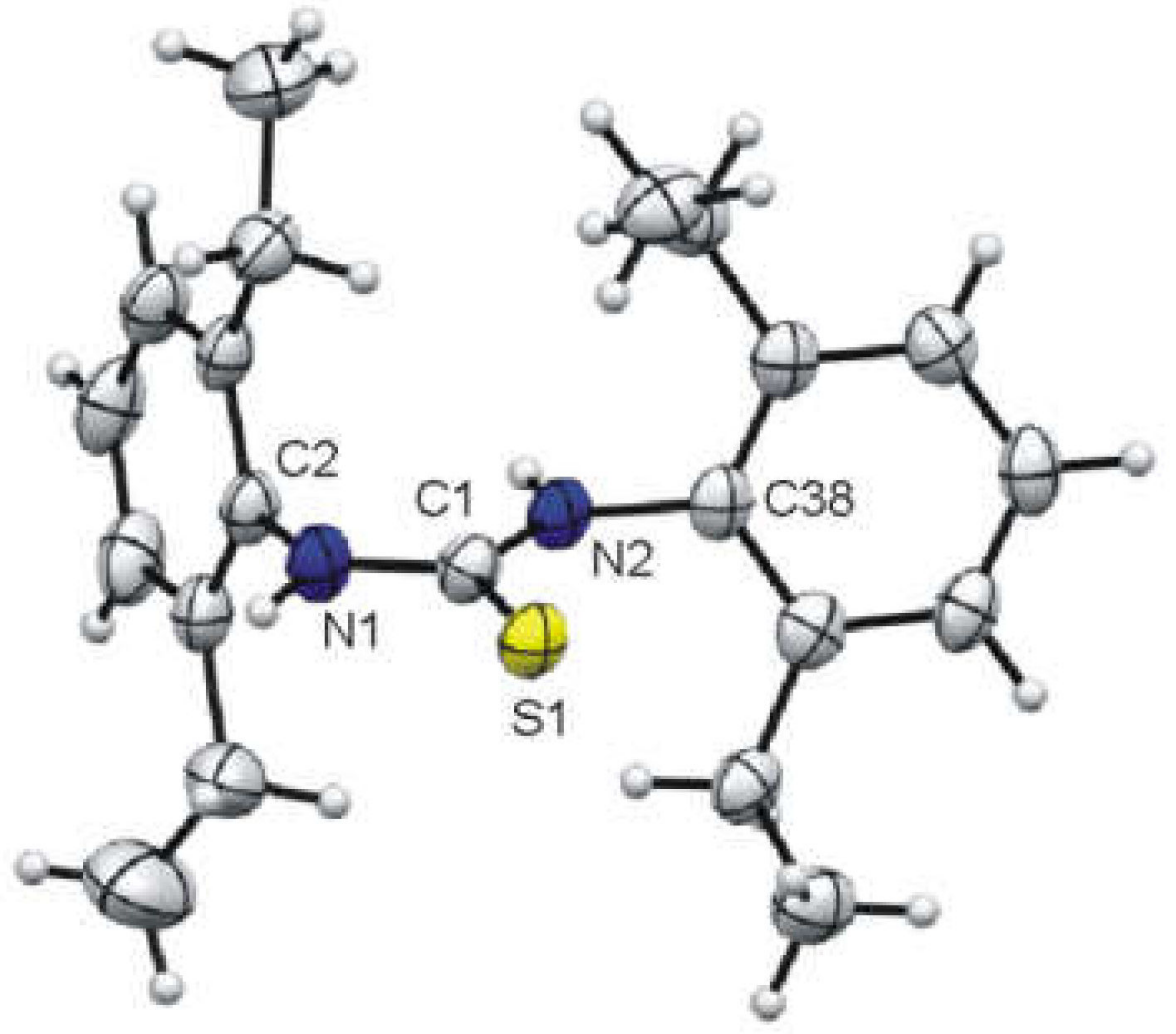
Molecular structure of 1,3-bis(2,6-diethylphenyl)thiourea ligand (ellipsoids correspond to the 50% probability level). Selected bond lengths (Å) and angles (°): S1–C1 1.682(3), N2–C1 1.342(3), N2–C38 1.437(4), C1–N11.350(4), C2–N1 1.432(3); C1–N2–C38 126.5(2), N2–C1–N1 116.2(2), N2–C1–S1 123.1(2), N1–C1–S1 120.7(2), C1–N1–C2 126.5(2).

**Figure 2:**
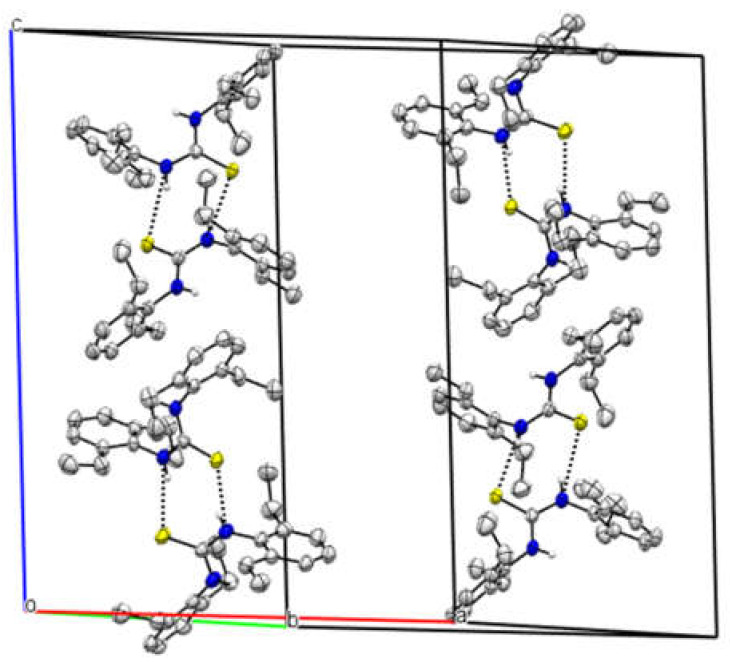
The partial packing figure of 1,3-bis(2,6-diethylphenyl)thiourea ligand along a, b and c axes (which shows the intermolecular H-bonds). Only N-H hydrogens are shown. Color scheme: S = yellow; N = blue; C = grey; and H = white.

Compound **L** crystallized in monoclinic crystal system in P2_1_/c space group with six molecules in the unit cell. The two aromatic groups are in *trans* position with respect to the thiourea unit (Figure 1). This configuration favors dimer formation between the molecules, and in this case, intermolecular N–H⋯S hydrogen bonds (3.266 and 3.328 Å) link the molecules into dimers (Figure 2). One of the ethyl groups shows disorder, and this is the reason for the relatively large displacement parameter and an unusual C8–C9 bond distance (1.428(6) Å). The short C–S distance (1.682(3) Å) clearly shows its double bond character (for comparison, C–S single bond is 1.82 Å) [57]. The S1–C1–N1–C2 and N2–C1–N1–C2 torsion angles are 1.58 and 178.42°, respectively. Experimental details of the X-ray single crystal structure analyses are summarized in Table 1.

**Scheme 1:**
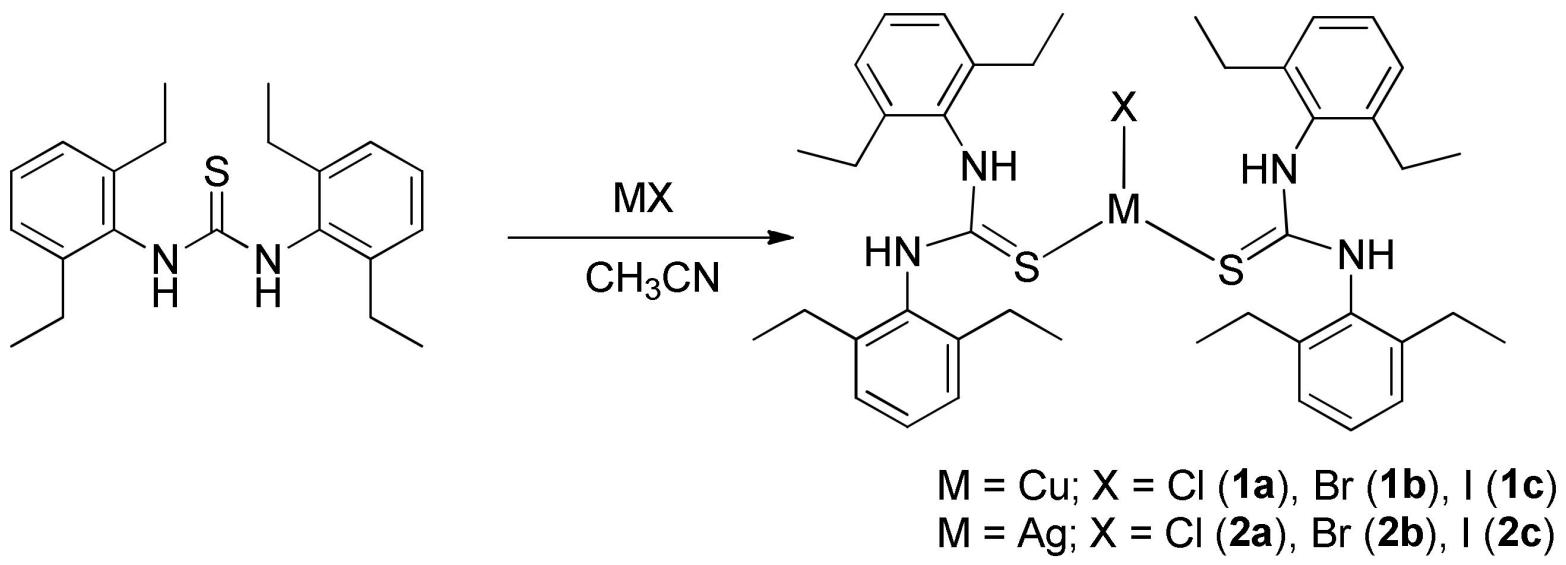
Syntheses of mononuclear tricoordinated copper (I) and silver (I) complexes of bulky 1,3-bis(2,6-diethylphenyl)thiourea ligand.

To isolate the metal complexes, acetonitrile was added to **L** and the corresponding metal (I) halide (metal = Cu, Ag; halide = Cl, Br, I) in 2:1 ratio at room temperature (Scheme 1). The reaction mixture was then stirred overnight at room temperature. After filtration, the filtrate was concentrated and stored at room temperature to afford the corresponding crystalline compounds [MXL_2_] [CuClL_2_].2CH_3_CN (**1a**); [CuBrL_2_].2CH_3_CN (**1b**); [CuIL_2_] (**1c**); [AgClL_2_] (**2a**); [AgBrL_2_] (**2b**); and [AgIL_2_] (**2c**) in high yields (74–94%). All the complexes were soluble in most common organic solvents, such as acetonitrile, acetone, methanol, methylene chloride, chloroform, and aqueous ethanol.

The ^1^H NMR spectra of **1** and **2** in CDCl_3_ exhibit resonances for N–H protons at higher and lower fields (δ = 6.35 and 10.52 ppm (**1a**), δ = 6.35 and 10.28 ppm (**1b**), and δ = 6.37 and 9.98 ppm (**1c**) and δ = 6.45; and 10.86 ppm (**2a**), δ = 6.43 and 10.0 ppm (**2b**), and δ = 6.43 and 9.31 ppm (**2c**), respectively). The shift to lower field could be attributed to the possible presence of intramolecular hydrogen bonding between thiourea and halide ligands [58,59]. For comparison, a singlet appears at 6.42 ppm in CDCl_3_ for the two N–H protons of the free ligand [21]. The ^13^C NMR spectra of **1** and **2** show characteristic peaks at 178.9 ppm (**1a**), 178.6 ppm (**1b**), and 178.0 ppm (**1c**); and 178.7 ppm (**2a**), 179.1 ppm (**2b**), and 179.7 ppm (**2c**), respectively, for C=S moiety (see Supplementary Materials). The other aliphatic and aromatic chemical shifts for both bulky thiourea ligands are in the typical regions with very less variation. The NMR data of **1a/b** show that these complexes are stable even if the co-crystallized acetonitrile molecules are removed by drying the samples in high vacuum.

All complexes except **2b** were further characterized by X-ray structural and elemental analyses. All compounds are mononuclear three-coordinated metal(I) halide complexes, in which metal is coordinated by two sulfur atoms of each bulky thiourea ligand, and one halogen atom, thus exhibiting trigonal planar molecular configurations with a butterfly structure. The driving force for this structure might be the intramolecular hydrogen bonds between halogen and the two nitrogen atoms of the two ligands to form pseudo-six-membered rings (NHClCuSC). Intermolecular hydrogen bonds observed were as a result of the solvated molecules. Experimental details of the X-ray single-crystal structure analyses are summarized in Table 1.

**Figure 3:**
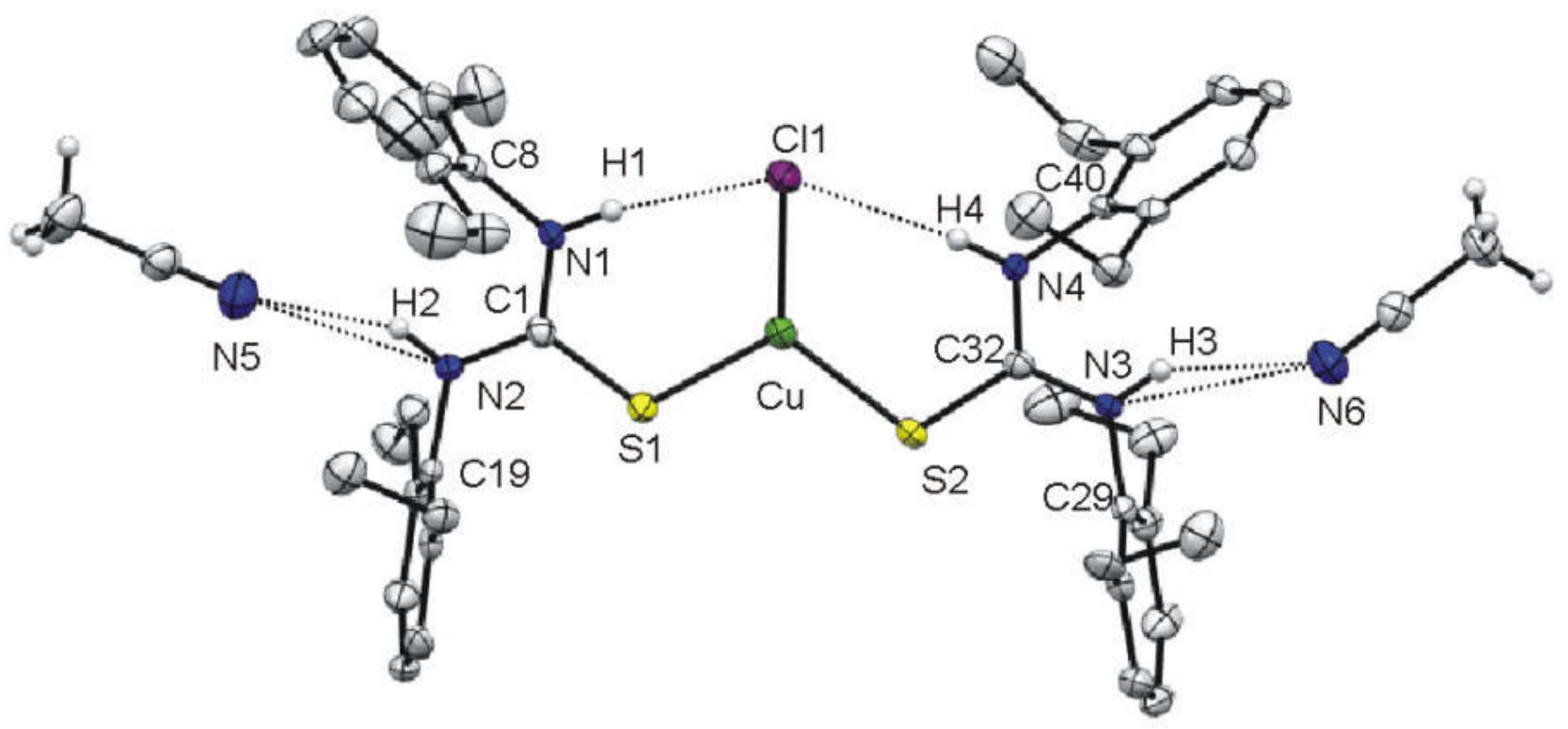
Molecular structure of **1a** (ellipsoids correspond to the 50% probability level). All hydrogen atoms, except those of N-H and CH_3_ hydrogens of acetonitrile, have been deleted for clarity. Selected bond lengths (Å) and angles (°): Cu1–S1 2.2176(9), Cu1–Cl1 2.2440(9), Cu1–S2 2.2180(9), S1–C11 1.708(3), S2–C32 1.710(3), N3–C29 1.452(3), N3–C32 1.330(4), N2–C19 1.454(3), N2–C11 1.331(4), N4–C40 1.445(3), N4–C32 1.345(3), N1–C8 1.434(4), N1–C11 1.341(3); S1–Cu1–Cl1 123.36(3), S1–Cu1–S2 110.34(3), S2–Cu1–Cl1 126.29(3), C11–S1–Cu1 110.75(10), C32–S2–Cu1 108.85(10), C32–N3–C29 124.2(2), C11–N2–C19 122.1(2), C32–N4–C40 122.8(2), C11–N1–C08 125.2(3), C18–C19–N2 118.4(2), C14–C19–N2 118.7(3), N2–C11–S1 119.9(2), N2–C11–N1 118.7(3), N1–C11–S1 121.4(2), N3–C32–S2 121.1(2), N3–C32–N4 118.0(3), N4–C32–S2 120.9(2).

**Figure 4:**
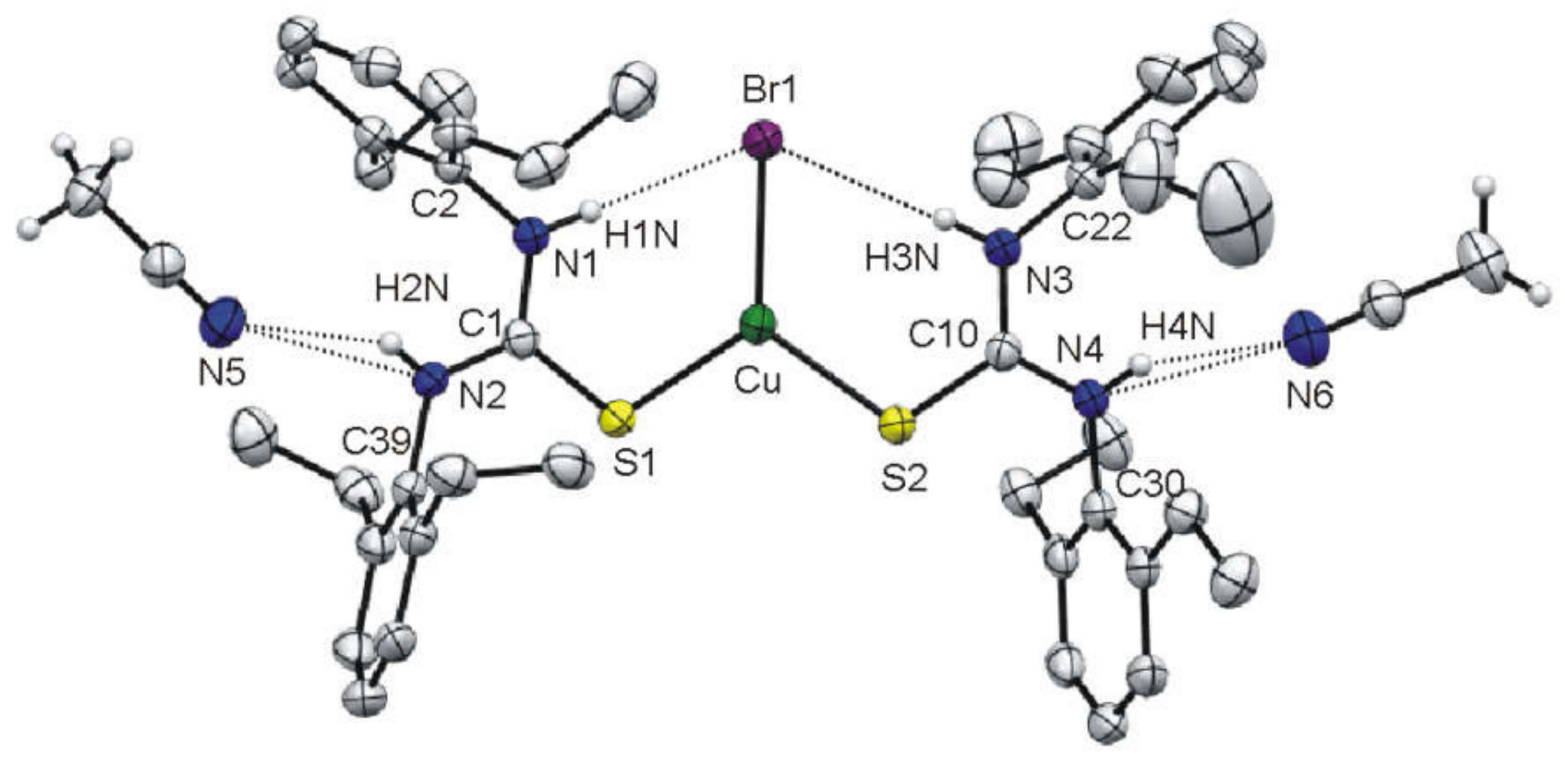
Molecular structure of **1b** (ellipsoids correspond to the 50% probability level). All hydrogen atoms, except those of N-H and CH_3_ hydrogens of acetonitrile, have been deleted for clarity. Selected bond lengths (Å) and angles (°): Br1–Cu1 2.3721(4), Cu1–S1 2.2143(7), Cu1–S2 2.2186(7), S1–C1 1.707(2), S2–C10 1.703(2), N1–C1 1.331(3), N1–C2 1.441(3), N3–C10 1.331(3), N3–C22 1.441(3), C1–N2 1.336(3), N2–C39 1.431(3), N4–C10 1.338(3) N4–C30 1.438(3); S1–Cu1-S2 111.40(3), S1–Cu1-Br1 125.56(2), S2–Cu1–Br1 123.04(2), C1–S1–Cu1 109.82(8), C10–S2–Cu1 110.94(8), C1–N1–C2 123.8(2), C10–N3–C22 125.1(2), N1–C1–N2 117.8(2), N1–C1–S1 121.96(18), N2–C1–S1 120.19(18), C1–N2–C39 125.0(2), C10–N4–C30 122.8(2).

**Figure 5:**
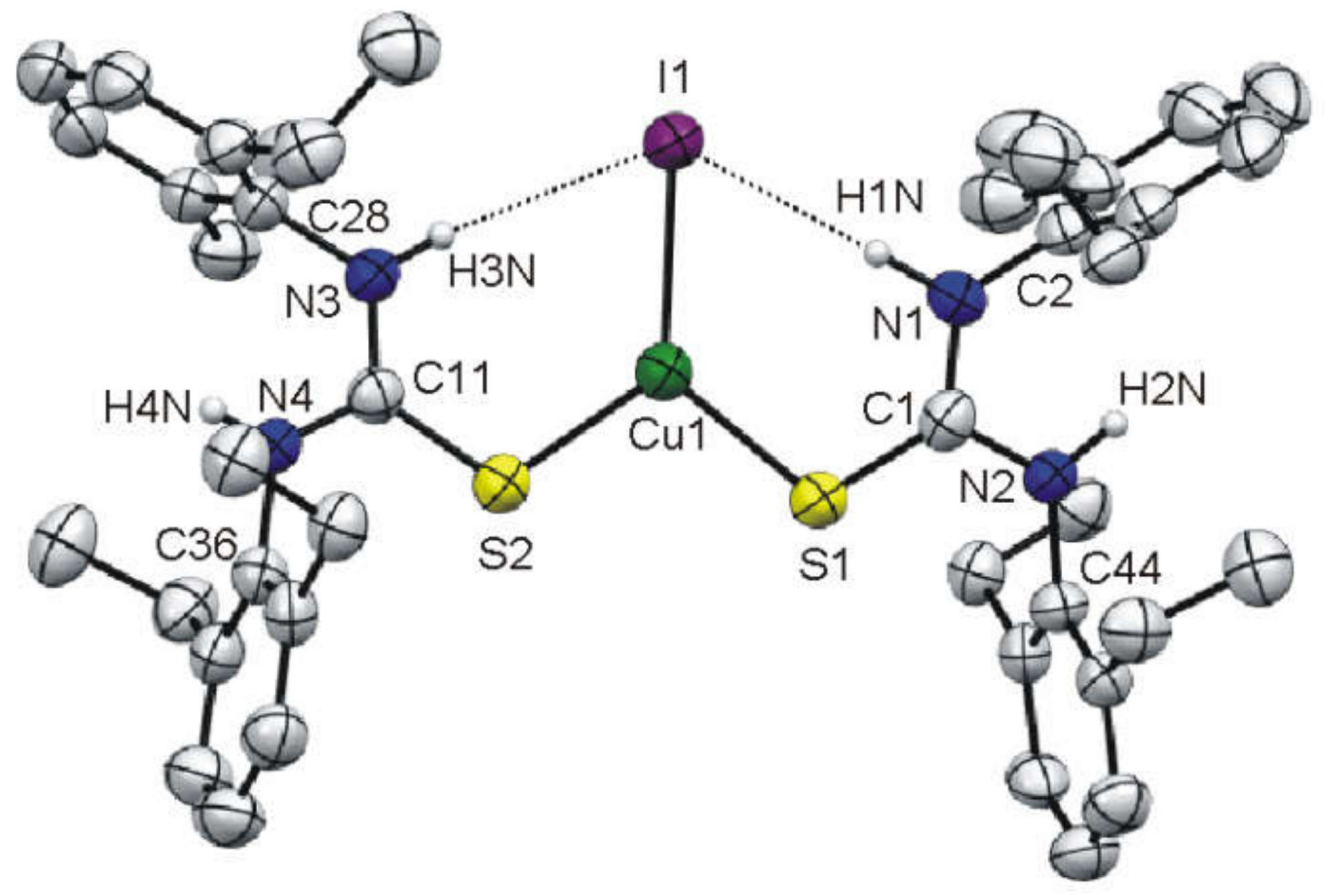
Molecular structure of **1c** (ellipsoids correspond to the 50% probability level). All hydrogen atoms, except those of N-H hydrogens and one molecule, have been deleted for clarity. Selected bond lengths (Å) and angles (°): I1–Cu1 2.5134(8), Cu1–S2 2.2197(15), Cu1–S1 2.2328(15), S1–C1 1.681(6), S2–C11 1.695(5), N2–C1 1.351(7), N2–C44 1.444(6), N3–C11 1.338(7), N3–C28 1.450(7), N1–C1 1.345(7), N1–C2 1.432(7), N4–C11 1.324(7), N4–C36 1.447(7); S2–Cu1–S1 108.03(6), S2–Cu1–I1 126.39(5), S1–Cu1–I1 125.57(5), C1–S1–Cu1 110.19(19), C11–S2–Cu1 111.91(18), C1–N2–C44 123.8(5), C11–N3–C28 122.9(5), C1–N1–C2 124.3(5), C11–N4–C36 125.1(5), N1–C1–N2 115.7(5), N1–C1–S1 123.2(4), N2–C1–S1 121.1(4), N4–C11–N3 117.4(5), N4–C11–S2 119.7(4), N3–C11–S2 122.9(4).

Compounds **1a** and **1b** both crystallize in a monoclinic crystal system with two CH_3_CN molecules each in the unit cells. Solvent-free copper complex **1c** crystalizes (from CHCl_3_) in triclinic space group P-1 with two molecules in the unit cell. In **1b**, one of the methyl of ethyl groups has relatively large displacement parameter due to disorder and is the reason for an unusual C21–C29 bond distance (1.386(7) Å). In all three copper compounds, the two intramolecular N–H⋯X (X = Cl, Br, I) hydrogen bonds complete the twisted six-membered rings (Figure 3, Figure 4 and Figure 5). In **1a**, the two intramolecular hydrogen bonds N1–H1⋯Cl1 (3.349 Å) and N4–H4⋯Cl1 (3.367 Å) are nearly identical but longer than those observed for similar 1,3-bis(2,6-dimethylphenyl)thiourea Cu(I) chloride complex [3]. In **1b** and **1c**, one of the two hydrogen bond’s D⋯A distance (Å) and D–H⋯A angle (°), N1–H1N⋯Br1 (3.464, 177) and N3–H3N⋯I1 (3.651, 175), are longer and wider than the other, N3–H3N⋯Br1 (3.393, 169) and N1–H1N⋯I1 (3.555, 163). The bond angles observed for **1b** are identical to those reported by Tahir and co-workers for similar *N*,*N*′-bis(diphenyl)thiourea complexes (3.435 (2) Å, 169° and 3.573 (2) Å, 170°) [60]. In **1a**, the Cu–Cl bond distance of 2.2440(9) Å is comprehensively shorter than the Cu–Cl bond distance (2.3058(10) Å) recently published by Nembenna and co-workers [8]. The Cu–Br bond distance of 2.3721(4) Å in **1b** is slightly longer than the Cu–Br bond distance in *N*,*N*′-bis(diphenyl)thiourea stabilized copper(I) complex [20] but comparable to *N*,*N*′-dicyclohexylthiourea stabilized copper complex (2.3801(5) Å) [61]. The slight increase in C=S bond distance of the bridging NCN moiety on binding with copper for **1a** and **1b** (average bond distance of 1.709 Å and 1.705 Å, respectively) compared to free ligand is common for such ligands. The shorter Cu–I (2.5134(8) Å) and Cu1–S1 (2.2328(15) and Cu1–S2 2.2197(15) Å) bond distances in **1c** than the previously known copper(I) iodide complexes could be attributed to the different coordination environment [62,63,64].

**Figure 6:**
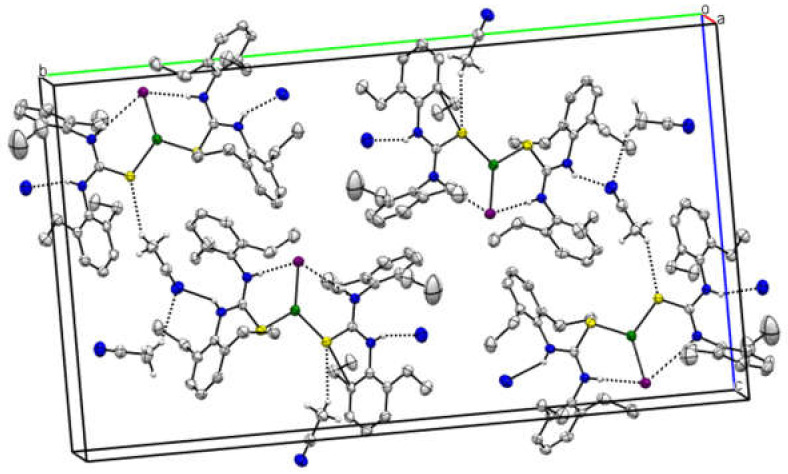
Perspective drawing of the packing arrangement of **1b** in the crystal along a, b and c axes. Only N-H and CH_3_ hydrogens of acetonitrile are shown. Color scheme: Cu = green; S = yellow; N = blue; Br = purple; C = grey; and H = white.

In **1a** and **1b**, the NH-groups not involved in H-bonding with halide-atom make intermolecular H-bonds with N-atoms of acetonitrile (N–H⋯N distances of 2.982 and 2.993 Å in **1a** and 2.988 and 2.980 Å in **1b**). It is interesting to see that two of the CH_3_-hydrogens of the acetonitrile are also involved in hydrogen bonding (one with S-atom of thiourea (C–H⋯S 3.664 Å in **1a** and 3.670 Å in **1b**) and the other with N-atom (C–H⋯N 3.517 Å in **1a** and 3.543 Å in **1b**) of another acetonitrile), and the third makes a π interaction (only **1b** is shown in Figure 6).

**Figure 7:**
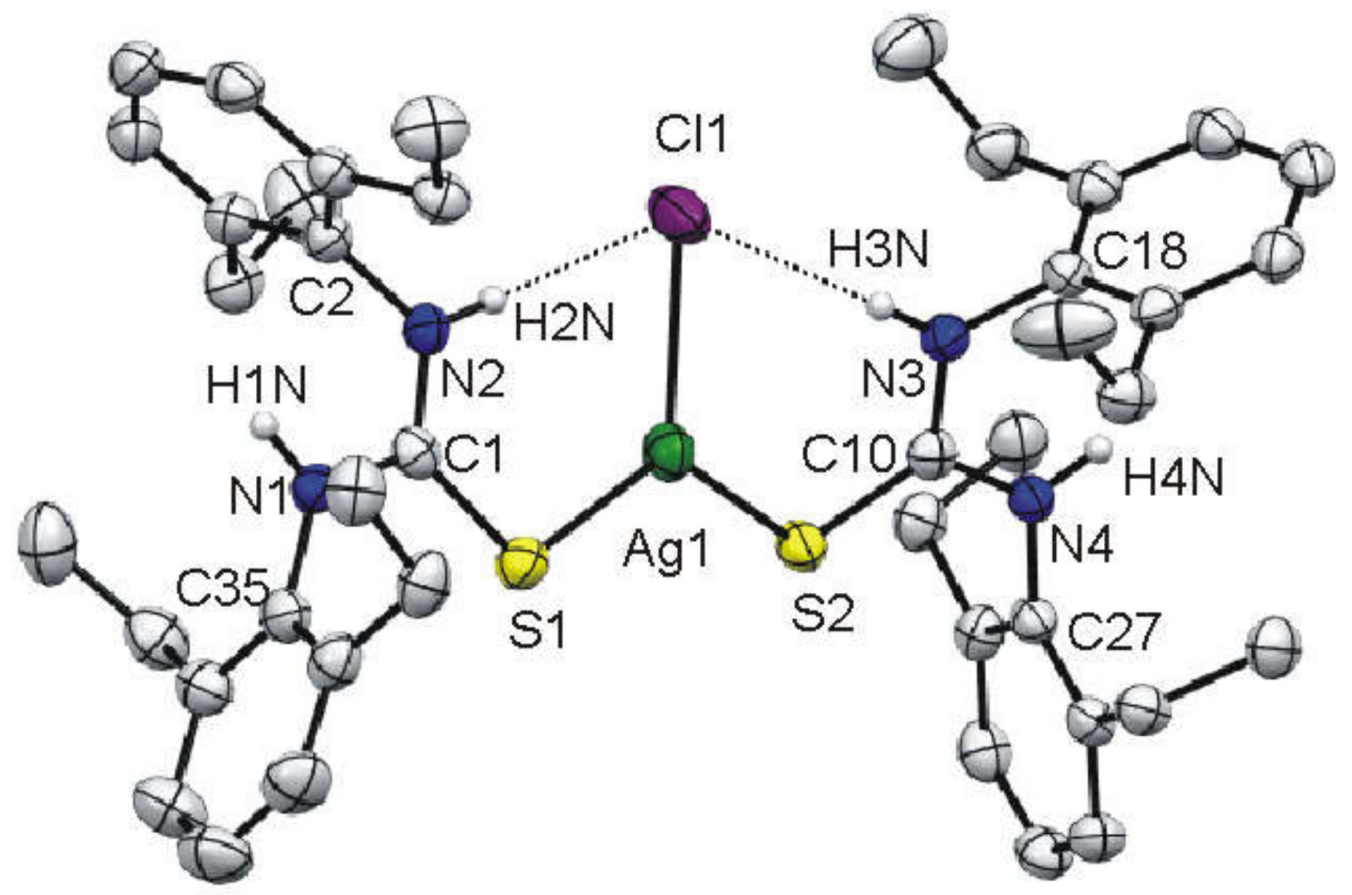
Molecular structure of **2a** (ellipsoids correspond to the 50% probability level). Hydrogen atoms, except those of N-H, have been deleted for clarity. Selected bond lengths (Å) and angles (°): Ag1–S2 2.4257(8), Ag1–S1 2.4362(9), Ag1–Cl1 2.5040(10), S2–C10 1.690(3), S1–C1 1.698(3); S2–Ag1–S1 126.79(3), S2–Ag1–Cl1 116.23(3), S1–Ag1–Cl1 116.94(3), C10–S2–Ag1 108.36(11), C1–S1–Ag1 106.02(11), C1–N1–C35 126.1(3), C10–N3–C18 120.7(3), C10–N4–C27 124.0(3), C1–N2–C2 122.4(3), N2–C1–N1 117.3(3), N2–C1–S1 122.8(2), N1–C1–S1 119.9(2), N4–C10–N3 116.4(3), N4–C10–S2 119.7(2),N3–C10–S2 123.9(2).

**Figure 8:**
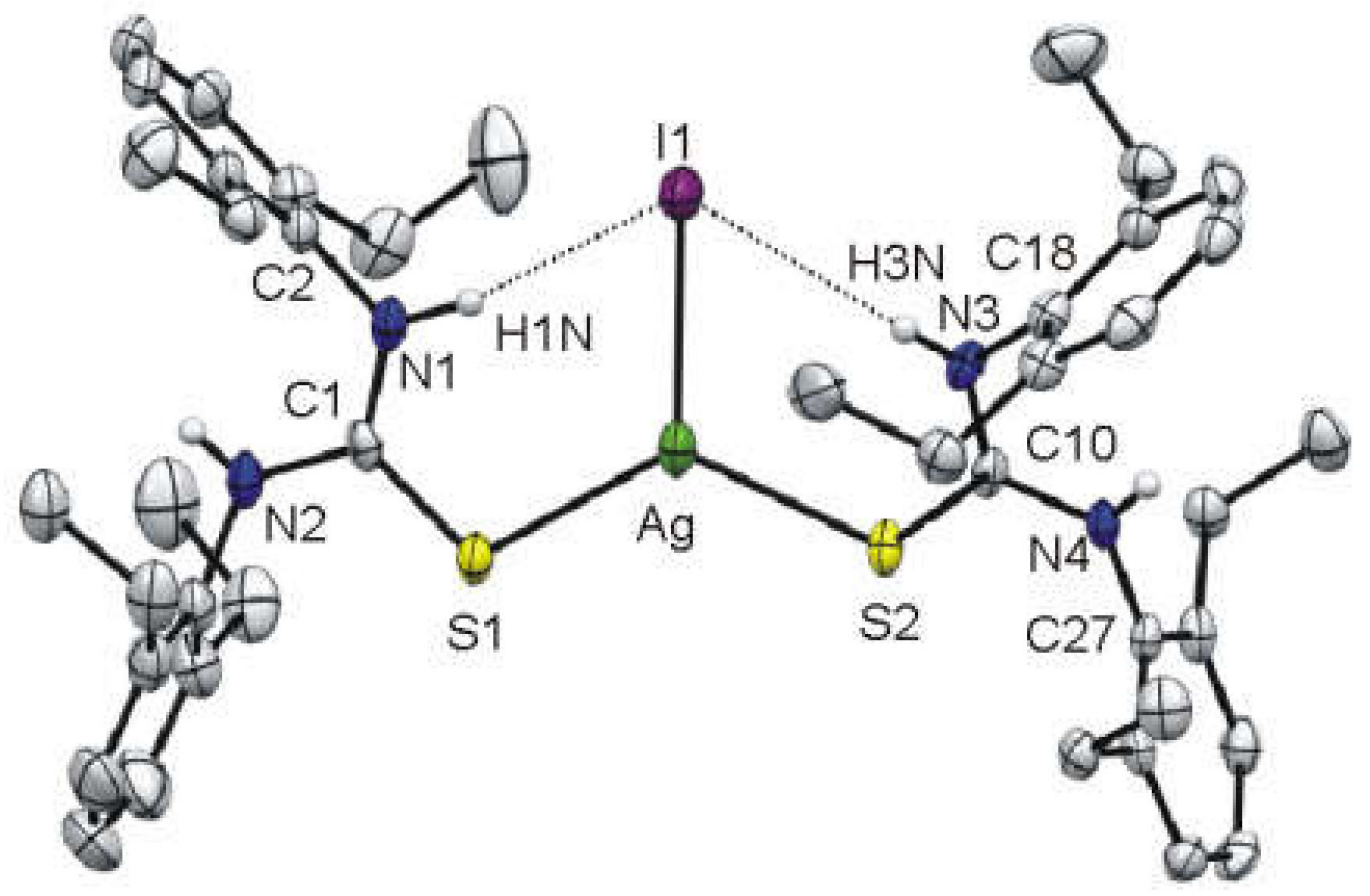
Molecular structure of **2c** (ellipsoids correspond to the 50% probability level). Hydrogen atoms, except those of N-H, have been deleted for clarity. Selected bond lengths (Å) and angles (°): Ag1–S2 2.4594(12), Ag1–S1 2.4673(12), Ag1–I1 2.7505(6), S2–C10 1.705(5), S1–C1 1.710(5); S2–Ag1–S1 125.09(5), S2–Ag1–I1 114.82(3), S1–Ag1–I1 120.00(3), C10–S2–Ag1 110.48(16), C1–S1–Ag1 107.09(16), C10–N3–C18 121.5(4), C10–N4–C27 125.0(4), C1–N1–C2 123.7(4), N1–C1–N2 117.4(4), N1–C1–S1 122.8(3), N2–C1–S1 119.9(4), C1–N2–C35 127.0(4), N3–C10–N4 117.0(4), N3–C10–S2 123.9(3), N4–C10–S2 119.1(4).

Similar to **1a** and **1b**, crystals of silver complex **2a** were also obtained from acetonitrile, but without solvated molecules, whereas crystals of **2c** were grown from chloroform. Both **2a**/**c** crystallize in a orthorhombic crystal system in Pbca space group and are the first examples of structurally characterized tricoordinated thiourea-stabilized monomeric silver halide complexes (Figure 7 and Figure 8) [65,66]. The longer intramolecular hydrogen bonds D⋯A distances in **2c** (N1–H1N⋯I1 3.543 Å and N3–H3N⋯I1 3.702 Å) compared to **2a** (N2–H2N⋯Cl1 3.278 Å and N3–H3N⋯Cl1 3.472 Å) are attributed to the larger size of the iodide ligand. Although there is some variation in the S–Ag–X pair of angles (S1–Ag1–Cl1 116.94(3)° and S2–Ag1–Cl1 116.23(3)°; S1–Ag1–I1 120.00(3)° and S2–Ag1–I1 114.82(3)°), the S–Ag–S angles are essentially identical (S2–Ag1–S1 126.79(3)° (**2a**) and S2–Ag1–S1 125.09(5)° (**2c**)). The Ag–S bond exhibits slight increase from chloride to iodide (Ag1–S1 2.4362(9) Å and Ag1–S2 2.4257(8) (**2a**) Å; Ag1–S1 2.4673(12) and Ag1–S2 2.4594(12) (**2c**) Å). The Ag–S bonds observed in **2a** are comparable to closely related tricoordinate silver(I) chloro complex of imidazolidine-2-thiones (2.439(1) Å) [67]. The increase in Ag–X (Ag1-Cl1 2.5040(10) Å and Ag1-I1 2.7505(6) Å) is attributed to the increased size of iodide ligand.

The use of coordination compounds as metal-based drugs is known to lower the blood sugar levels in diabetic patients [68]. To develop a new drug, the fundamental quest is to understand their interaction and efficiencies with the receptor site. The availability of the 3D structure of the target protein makes molecular docking one of the best screen options. Molecular docking simulation was performed in order to evaluate the binding pattern of the synthesized metal complexes inside the active site of the human insulin (HI), using the default settings built in the MOE modelling tool.

**Figure 9:**
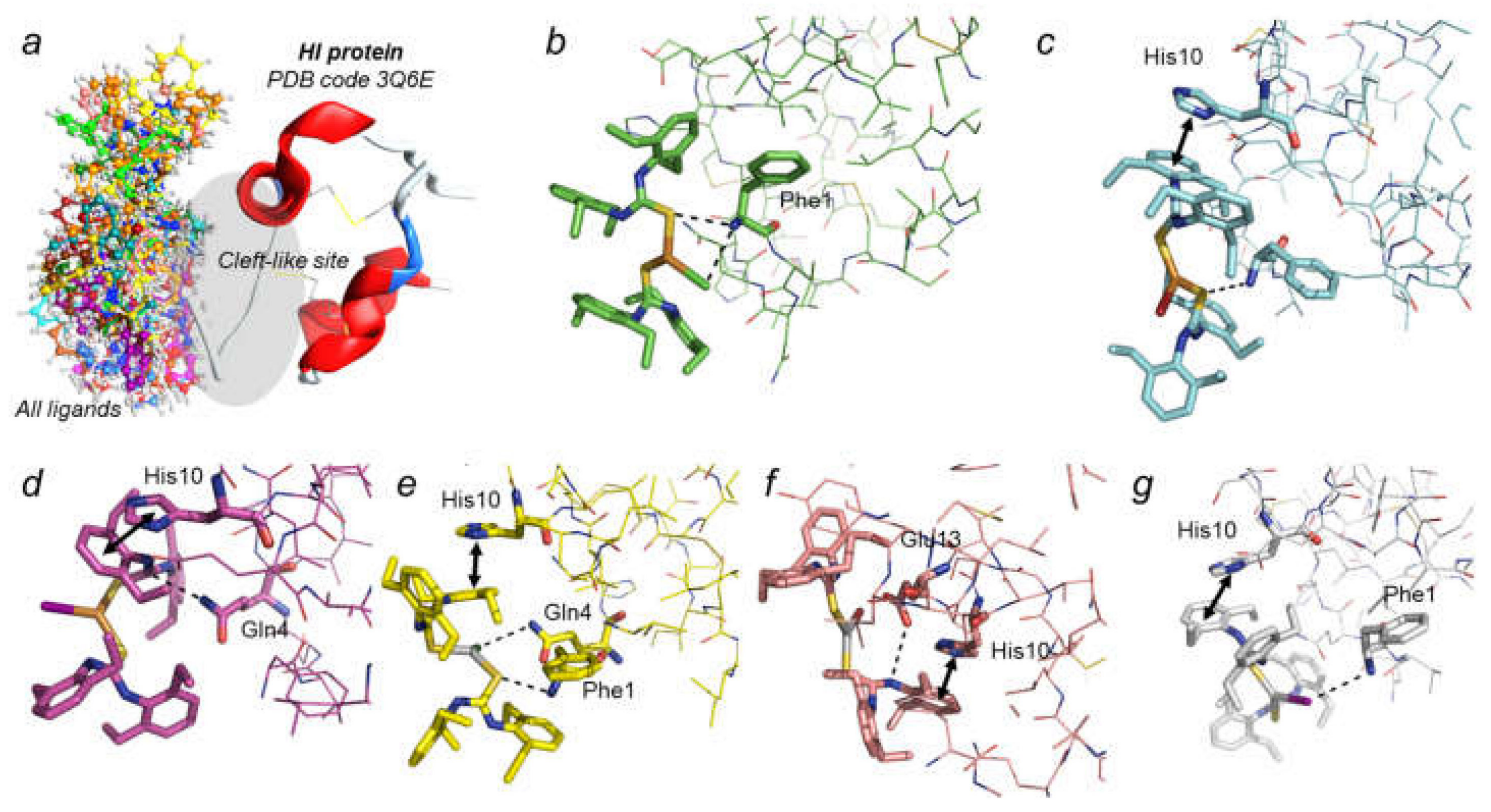
Analysis of the binding pattern of all the metal-based ligands complexes inside the active cleft of human insulin. (**a**) represents all the superposed docked metal-based ligands complexes; (**b**–**g**) represent the interaction profile for metal-based ligand complexes: (**b**) for **1a**, (**c**) for **1b**, (**d**) for **1c**, (**e**) for **2a**, (**f**) for **2b**, and (**g**) for **2c**. The molecule and interactive residues are shown as sticks, while the rest of the active site residues are shown in wireframe models. Hydrogen bonding interactions are shown as black dotted lines, metered with the distance in Å, and π–π interactions are shown by double-sided arrows.

In general, hydrophobic residues have been found to have a strong interaction with the ligand essential moiety. The best docking positions with the most interactions were those that were ranked first based on the least amount of energy (calculated as a negative value by MOE), as listed in Table 2. In order to further study the interactions of the metal-based docked conformations with the key residues, the most favorable docking poses of the 50 docked conformations for each metal complex were analyzed. Both the hydrophobic and hydrophilic portions were used to create the active site cleft (see in Figure 9a). Catalytic residues are mostly found in the hydrophilic region of the HI protein, and they may play an effective role in increasing or decreasing its activity.

Molecular docking studies showed that the metal complexes stabilize themselves inside the active site cleft through electrostatic and aromatic interactions. The N-terminal Phe and His residues may stabilize the molecules and prevent their dissociation from the active site residues. The hydrophobic and hydrophilic amino acid residues formed a stable host–guest complex by interacting via π-stacking and hydrogen bonding. The protein–ligand interaction profiles of the complexes are shown in Figure 9b–g. In all the complexes, the sulfur atom of the thiourea ligand was ligated with the corresponding metal atom of the complexes. Despite the rigid phenyl ring, all the complexes managed to attain the docking table conformation inside the active site cleft, as shown in Figure 9a. In addition to that, the phenyl established significant aromatic interactions with the active site residues, which played a significant role in anchoring the metal complexes inside the pocket of HI. The present docking study found that the silver-based complexes showed the strongest inhibitory potential against the HI protein, which possesses a deactivating group that is directly connected to silver. The deactivating group might withdraw some of the electronic density from the reaction center, leaving the moiety with a partial positive charge, which drives the moiety to form crucial contacts with critical residues, resulting in greater stability. Metal complexes **1a** and **2a**, containing chlorine, both exhibited interactions with the active site residue through the halogen atom, which can be attributed to the higher electronegative nature of chloride ion. Complexes having bromine and iodine did not show any type of electrostatic interactions with the active site residues. Thus, it can be argued that the electronegativity may play an important role in designing novel metal-based HI inhibitors. Since the halogen group withdraws electronic density from the compound, it needs to stabilize itself by adopting interaction with the active residues. In general, the hydrophobic residues preferred to establish the interactions with the important phenyl moiety of the complex. Aromatic moieties adopted interactions particularly with the sulfur and metal atoms, directly in the metal-based complexes. Interestingly, these hydrophobic residues bonded with high potential, and anchored the complex inside the active site of the protein with binding energy of around −10.7 to −7.1 kcal/mol, which is quite significant for stabilization of the metal complex inside the active site cavity. The impact of withdrawing the electronic density from the compound further compelled the compound to manage possible interaction for the ease of gaining a stable environment inside the active site cleft.

**Figure 10:**
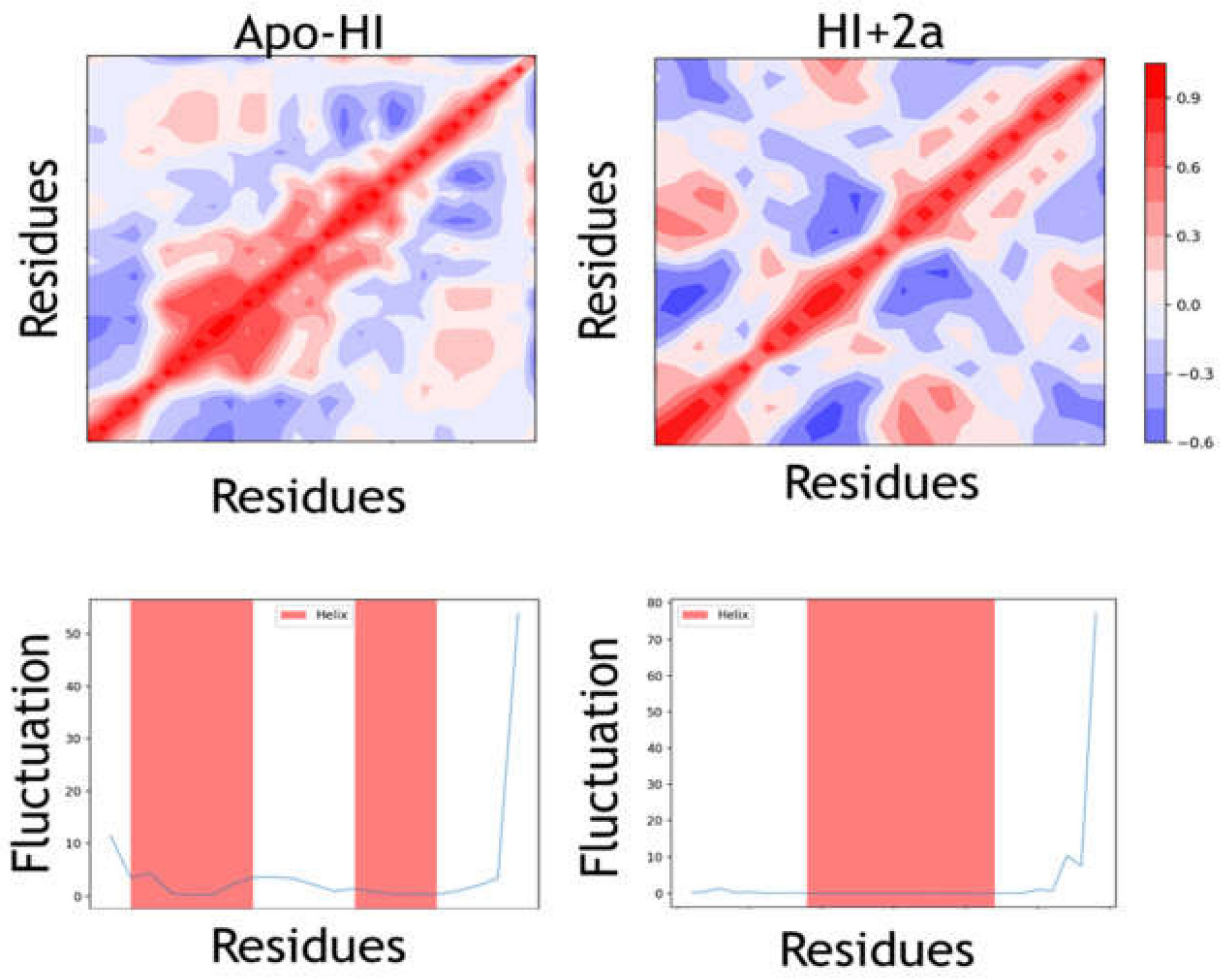
Normal mode analysis of **2a**. The upper panel (right with **2a** and left) indicates the correlation. The color bar indicates the positive (red) and negative (blue color) correlation. A stronger correlation indicates a more compact structure, while a negative correlation indicates a move-away behavior, which subsequently increases the plasticity of the structure, where it would be very easy for any compound to dissociate. The lower panel indicates the residue fluctuation.

Additionally, we have evaluated the normal mode analysis (NMA) for **2a** in order to check the dynamic nature/equilibrium modes of the complex (Figure 10). The NMA can model amino acids using Cα atoms to reduce computing cost. Normal mode analysis shows that the **2a** and protein complex is stabilized by harmonic potentials. The results indicate that upon binding with **2a**, the overall protein conformation remains compact and had a strong positive correlation instead of negative correlation, which further indicates that this complex remains in the active site and will need enough energy to be dissociated from the pocket. It was observed that the residues’ fluctuation is much higher than that of the complex state, revealing the impact of the complex in the binding site, to rescue the protein from high conformation. Thus, it can be suggested that these complexes might be used as a stand-in surrogate for developing and designing novel drugs.

## 4. Conclusions

The reaction of salts of univalent metals MX (where M = Cu or Ag and X = Cl, Br, and I) with sterically hindered thiourea ligand leads selectively to corresponding monomeric MXL_2_ complexes. All compounds are neutral, and air and moisture stable. Intermolecular H-bonds were responsible for forming dimers in free ligand. The tricoordinate monomeric complexes of silver halides reported here are the first structurally characterized examples of the vastly studied thiourea ligands. Intramolecular H-bonds were observed between N–H⋯X atoms, whereas intermolecular H-bonds were evident between solvated acetonitrile and metal-coordinated thiourea ligands. The docking results demonstrated that the silver-based ligand complex **2a** has a strong inhibitory potential against the HI protein, which contains a deactivating group that is directly linked to silver. Based on the current docking simulations, it is suggested that these complexes may further be investigated for their inhibitory potential against the HI. These may serve as a surrogate for discovery of novel HI inhibitors.

## Figures and Tables

**Table 1:** Crystallographic data for **L**, **1a**, **1b**, **1c**, **2a,** and **2c**.

Compound	L	1a	1b	1c	2a	2c
Empirical formula	C_21_H_28_N_2_S	C_46_H_62_ClCuN_6_S_2_	C_46_H_62_BrCuN_6_S_2_	C_42_H_56_CuIN_4_S_2_	C_42_H_56_AgClN_4_S_2_	C_42_H_56_AgIN_4_S_2_
Formula weight	340.51	862.176	906.58	871.46	824.34	915.79
Crystal system	monoclinic	monoclinic	monoclinic	Triclinic	Orthorhombic	Orthorhombic
Space group	*P*2_1_*/c*1	*P*2_1_/*n*	*P*2_1_/*n*	*P*-1	*P b c a*	*P b c a*
*a* (Å)	18.370(4)	8.1700(16)	8.1490(6)	13.0940(6)	18.532(4)	18.8900(6)
*b* (Å)	27.610(6)	31.440(6)	31.597(2)	15.2930(7)	15.377(3)	15.5390(4)
*c* (Å)	23.010(5)	17.850(4)	17.9060(12)	22.5210(11)	28.753(6)	28.7440(8)
*α* (deg)				80.430(4)		
*β* (deg)	93.30(3)	90.50(3)	90.282(5)	80.507(4)		
*γ* (deg)				72.124(4)		
V (Å^3^)	11,651(4)	4584.9(16)	4610.5(6)	4201.0(4)	8193(3)	8437.3(4)
Crystal size (mm^3^)	0.80 × 0.71 × 0.51	0.21 × 0.08 × 0.06	0.77 × 0.56 × 0.25	0.25 × 0.18 × 0.14	0.26 × 0.24 × 0.15	0.16 × 0.12 × 0.08
*ρ*_calcd_ (g cm^−3^)	1.165	1.249	1.306	1.378	1.337	1.442
*µ* (mm^−1^) (Mo K*α*)	0.171	0.664	1.469	1.387	0.693	1.342
*T* (K)	133(2)	293(2)	133(2)	133(2)	133(2)	133(2)
2θ range (deg)	4.33–59.07	4.56–55.13	2.57–53.28	2.82–51.17	2.20–53.37	3.67–57.39
No. of reflections unique	29,725	10,751	9242	16,774	8220	10,515
No. of reflections obs. (*I* > 2σ (*I*))	16,193	5400	7002	7906	4862	7605
No. of parameters	1321	523	531	949	459	475
w*R*^2^ (all data)	0.2338	0.0961	0.0995	0.1310	0.1043	0.1613
*R* value (*I* > 2σ (*I*))	0.0727	0.0480	0.0385	0.0487	0.0403	0.0561

**Table 2:** The interaction network for metal-based ligand complexes.

Compound	Ligand	Receptor	Interaction	Distance	E (kcal/mol)	Docking Score
**1a**	Cl S34	N PHE 1 N PHE 1	H-acceptor H-acceptor	3.42 3.34	−10.7 −4.5	−5.60408545
**1b**	S34 6-ring	N PHE 1 5-ring HIS 10	H-acceptor pi-pi	3.79 3.50	−4.9 −0.0	−5.06169271
**1c**	C 6 6-ring	NE2 GLN 4 5-ring HIS 10	H-acceptor pi-pi	3.43 3.77	−0.5 −0.0	−4.67316198
**2a**	Cl 5 S34 6-ring 6-ring	NE2 GLN 4 N PHE 1 N PHE 1 5-ring HIS 10	H-acceptor H-acceptor pi-cation pi-pi	3.74 3.38 3.33 3.75	−1.6 −5.8 −2.7 −0.0	−6.59288883
**2b**	N 38 6-ring	OE2 GLU 13 5-ring HIS 10	H-donor pi-pi	3.16 3.45	−0.7 −0.0	−5.93951702
**2c**	I 5 6-ring	N PHE 1 5-ring HIS 10	H-acceptor pi-pi	4.19 3.93	−7.1 −0.0	−5.07392883

## Data Availability

Supplementary crystallographic data can be obtained online free of charge via http://www.ccdc.cam.ac.uk/conts/retrieving.html (accessed on 17 June 2022) (or from the Cambridge Crystallographic Data Centre, 12 Union Road, Cambridge CB2 1EZ, UK; Fax: +44-1223-336-033; e-mail: deposit@ccdc.cam.ac.uk), deposition numbers CCDC-2161943 (**L**), CCDC-2161944 (**1a**), CCDC-2161948 (**1b**), CCDC-2161945 (**1c**), CCDC-2161946 (**2a**), and 2161947 (**2c**).

## Supplementary Materials

The following supporting information can be downloaded at: https://www.mdpi.com/article/10.3390/molecules27134231/s1, Figures S1–S12: 1H and 13C NMR spectra of compounds **1a**, **1b**, **1c**, **2a**, **2b** and **2c**.

## Sample Availability

Samples of the compounds **1a**, **1b**, **1c**, **2a**, **2b** and **2c** are available from the authors.
