# Supplementary material for: Mononuclear Tricoordinate Copper(I) and Silver(I) Halide Complexes of a Sterically Bulky Thiourea Ligand and a Computational Insight of Their Interaction with Human Insulin
Source: Molecules. 2022 Jun 30;27(13):4231. doi: 10.3390/molecules27134231 (PMC9268019; doi:10.3390/molecules27134231)
Supplement: Supplementary file 1 [file molecules-27-04231-s001.zip › molecules-1779035-supplementary.pdf]

Supporting Information

Mononuclear Tricoordinate Copper(I) and Silver(I) Halide Complexes of a Sterically Bulky Thiourea Ligand and a Computational Insight of their Interaction with Human Insulin

Awal Noor [1,*], Sadaf Qayyum [1], Farukh Jabeen [2] and Ashfaq Ur Rehman [3]

[1] Department of Basic Sciences, Preparatory Year Deanship, King Faisal University, Al-Hassa 31982, Saudi Arabia; sqayyum@kfu.edu.sa
[2] Department of Chemistry and Biochemistry, Laurentian University, 935 Ramsey Lake Road, Sudbury, ON P3E 2C6, Canada; fjabeen@laurentian.ca
[3] Department of Molecular Biology and Biochemistry, University of California, Irvine, CA 92697-3900, USA; aurehman@uci.edu
* Correspondence: anoor@kfu.edu.sa

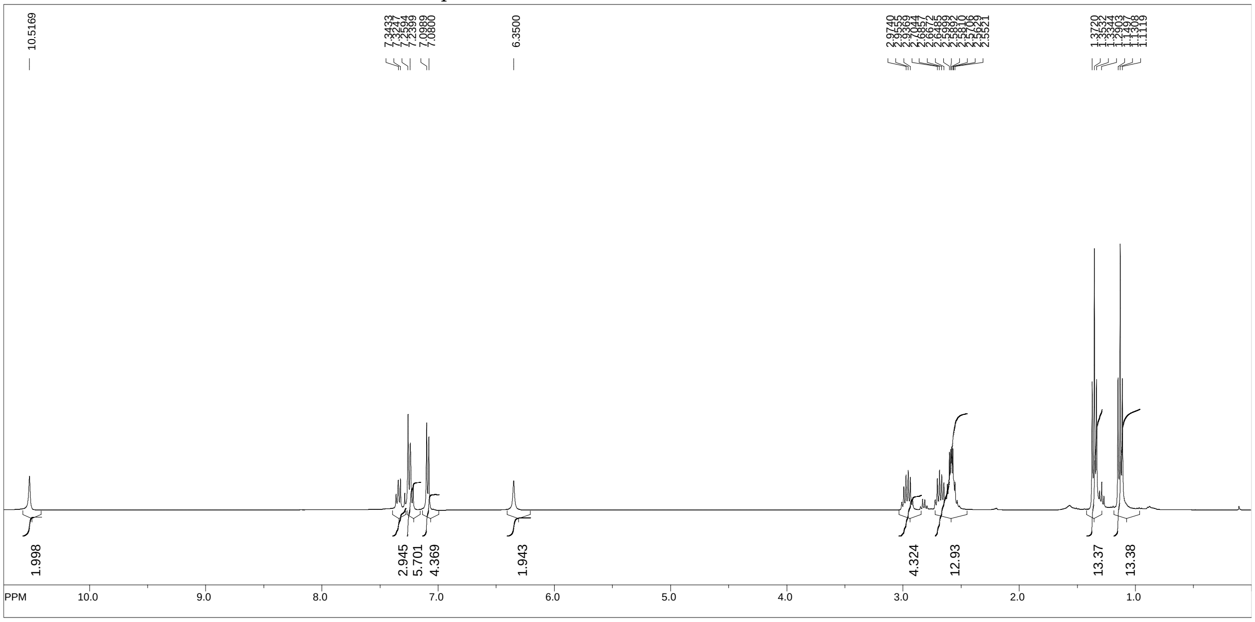

Figure S1. ^{1}H NMR spectrum of **1a**.

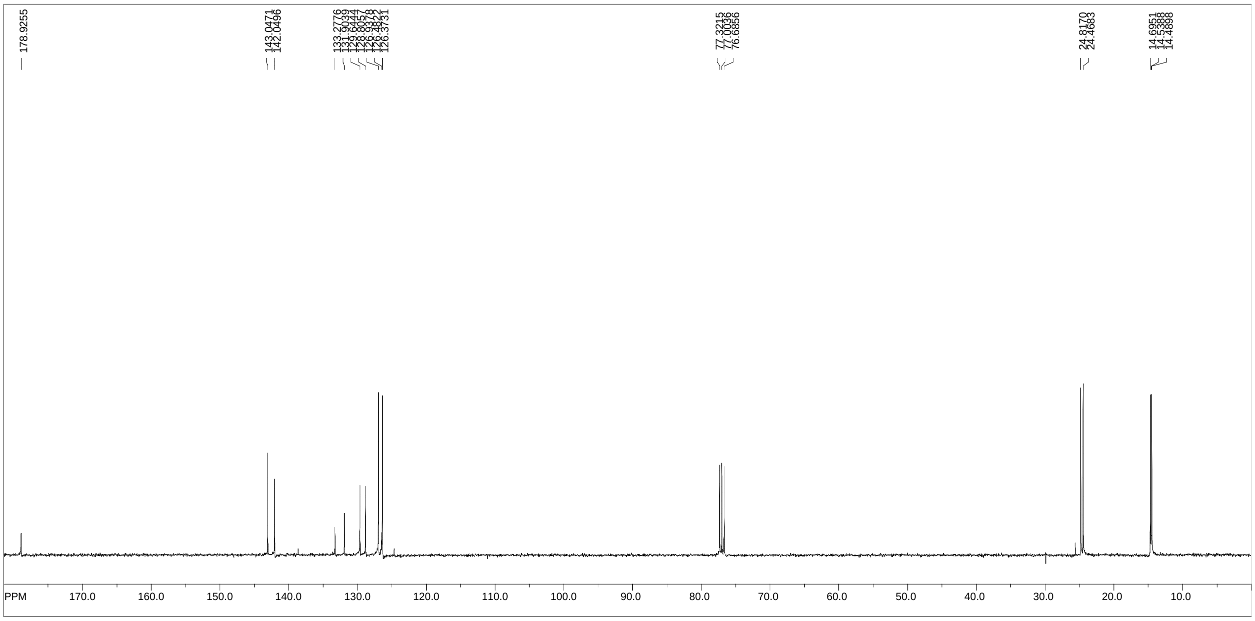

Figure S2. ^{13}C NMR spectrum of **1a**.

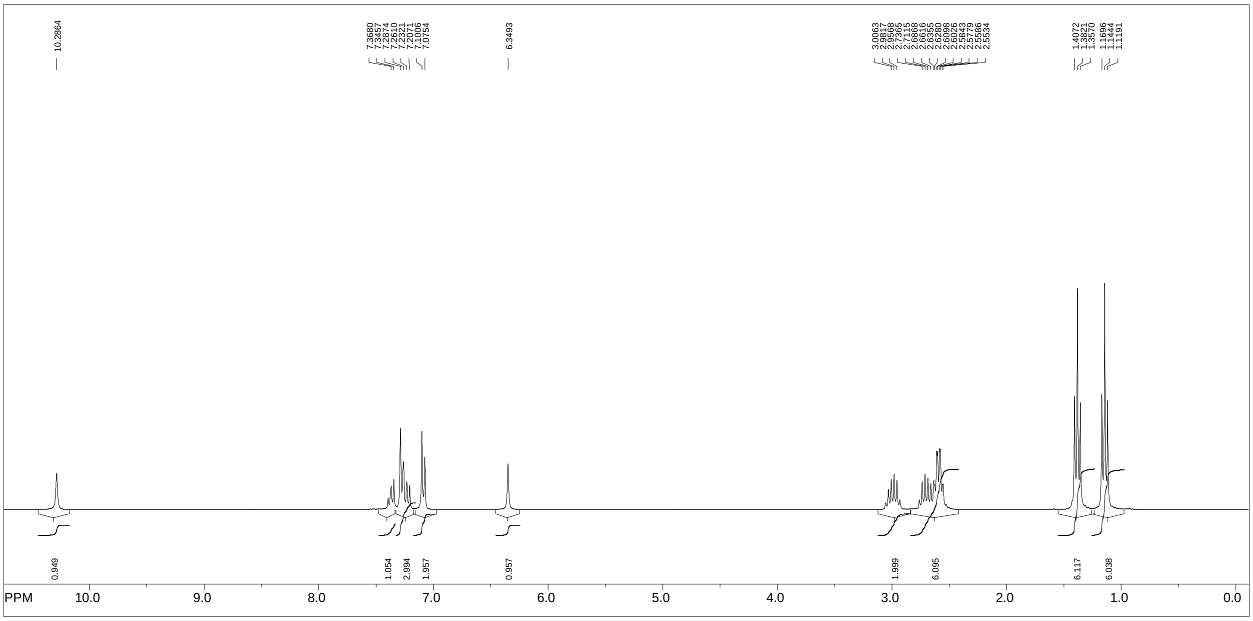

Figure S3. ^{1}H NMR spectrum of **1b**.

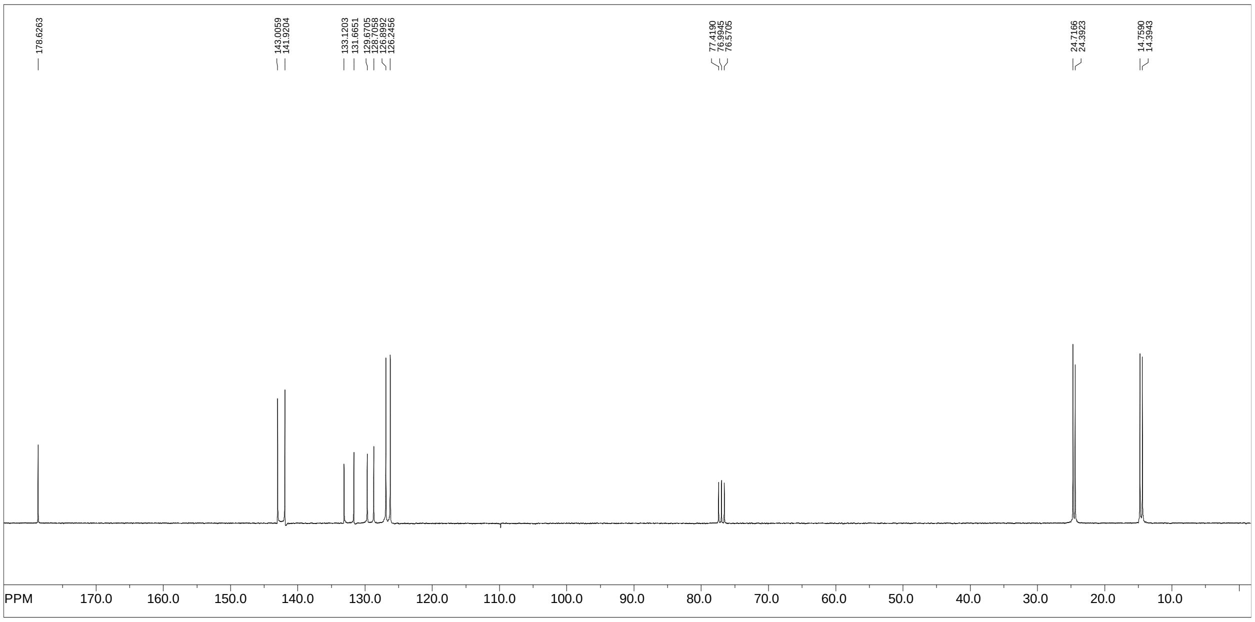

Figure S4. ^{13}C NMR spectrum of **1b.**

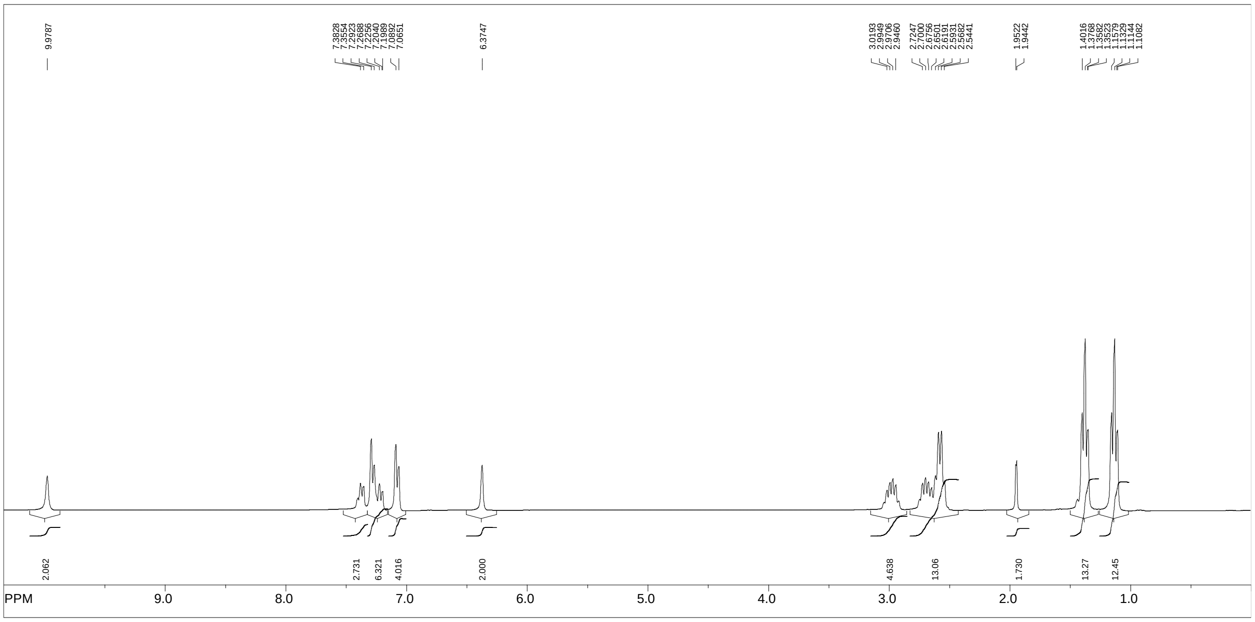

Figure S5. ^{1}H NMR spectrum of **1c**.

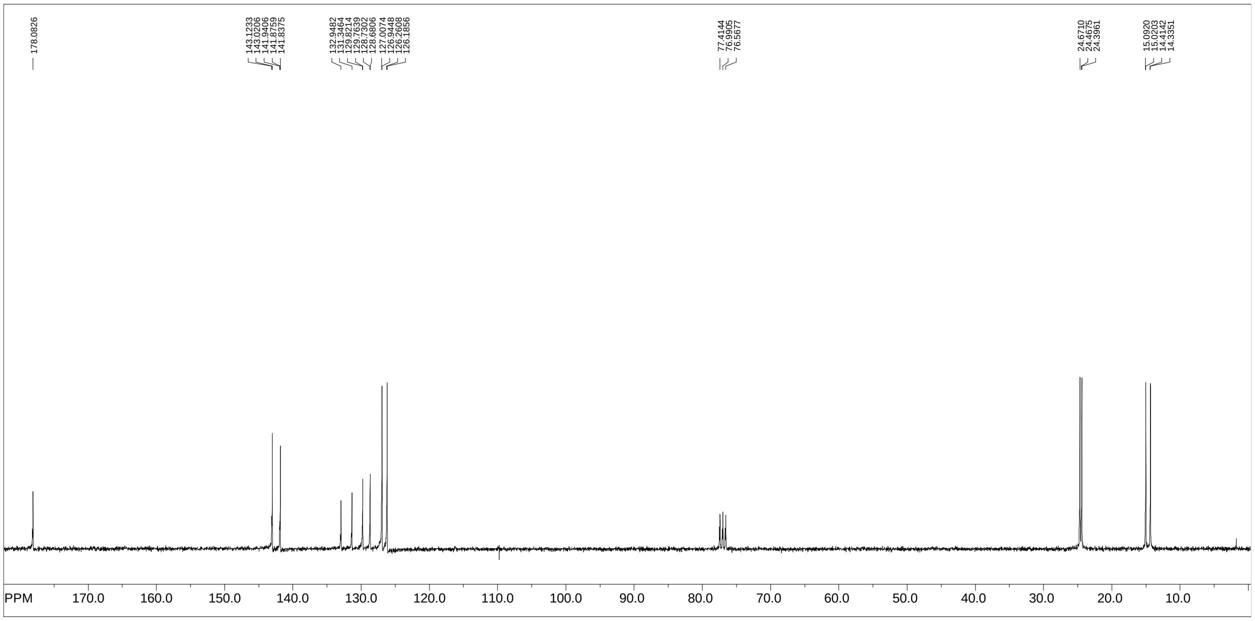

Figure S6. ^{13}C NMR spectrum of **1c**.

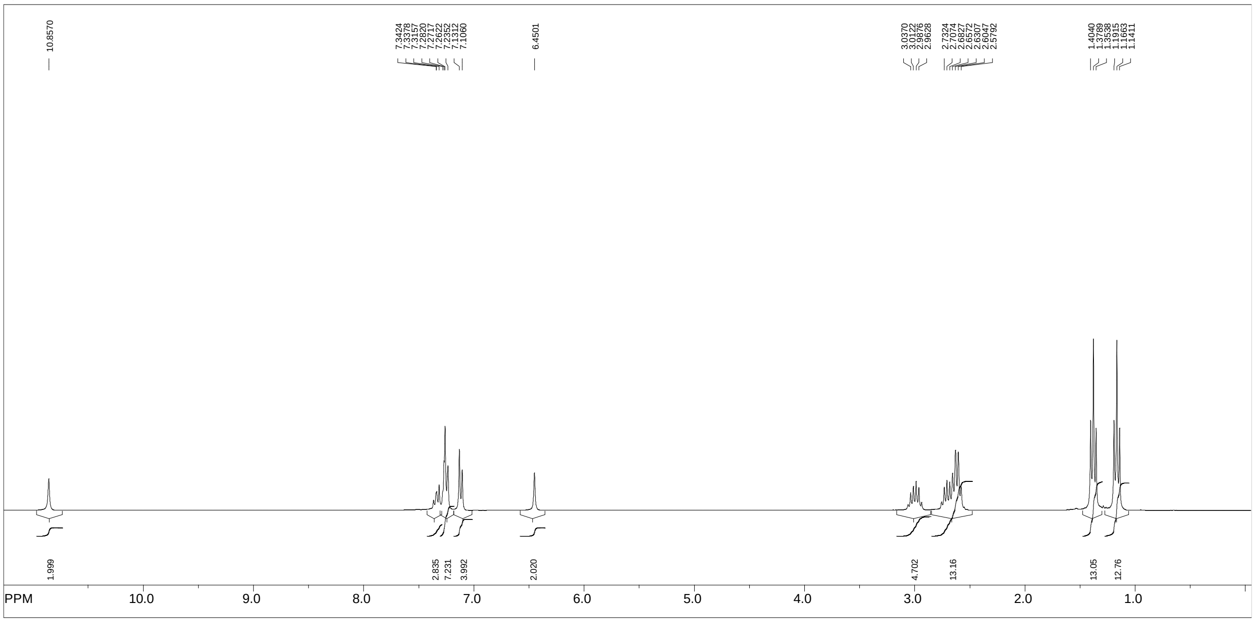

Figure S7. ^{1}H NMR spectrum of **2a**.

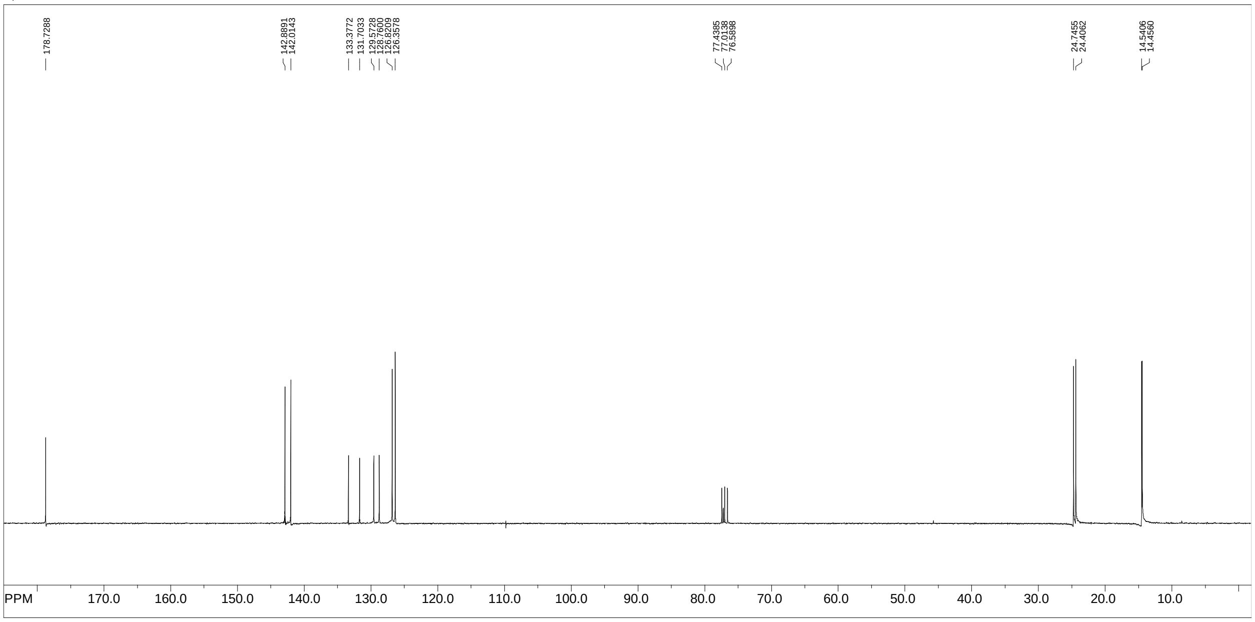

Figure S8. ^{13}C NMR spectrum of **2a**.

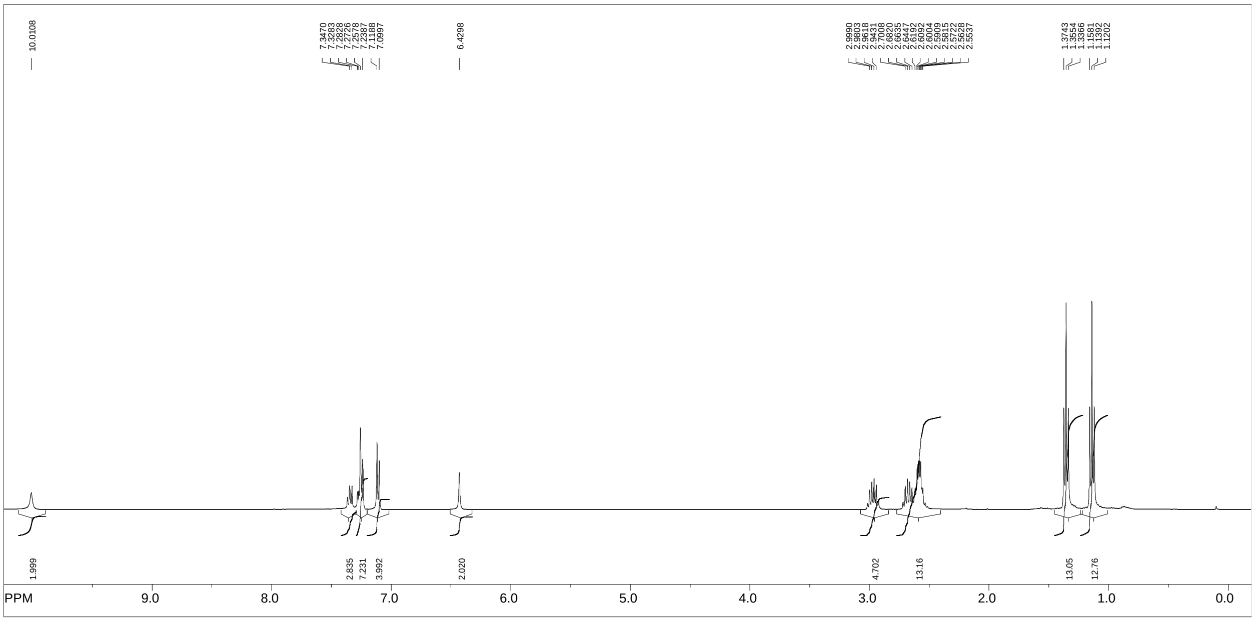

Figure S9. ^{1}H NMR spectrum of **2b**.

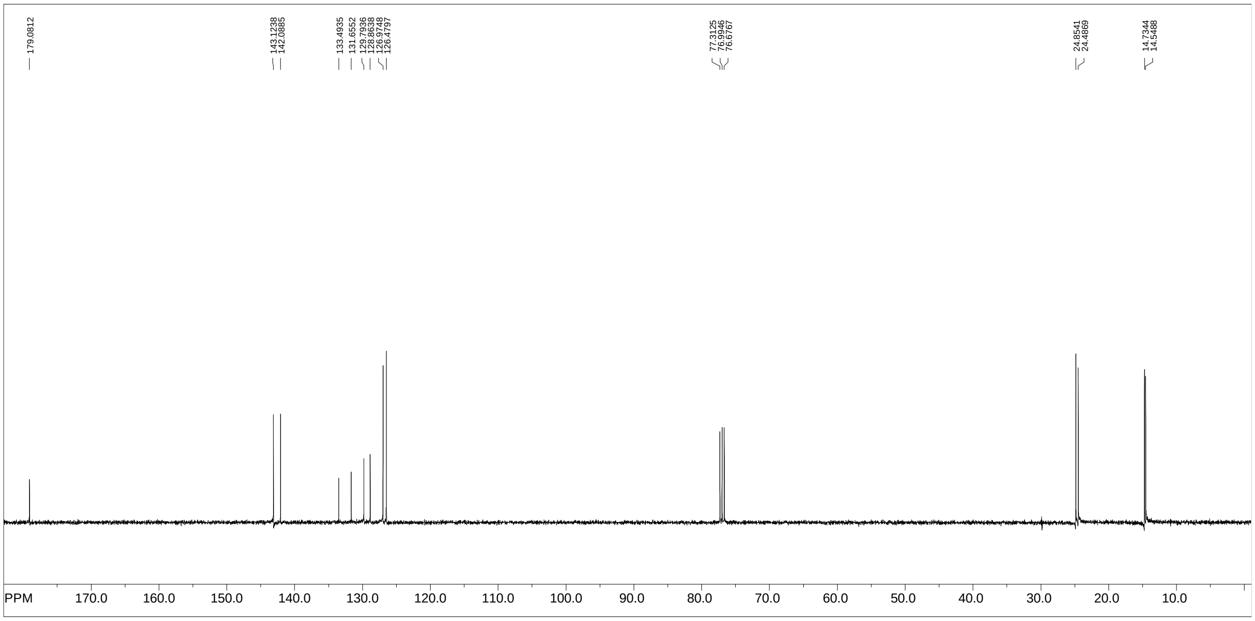

Figure S10. ^{13}C NMR spectrum of **2b**.

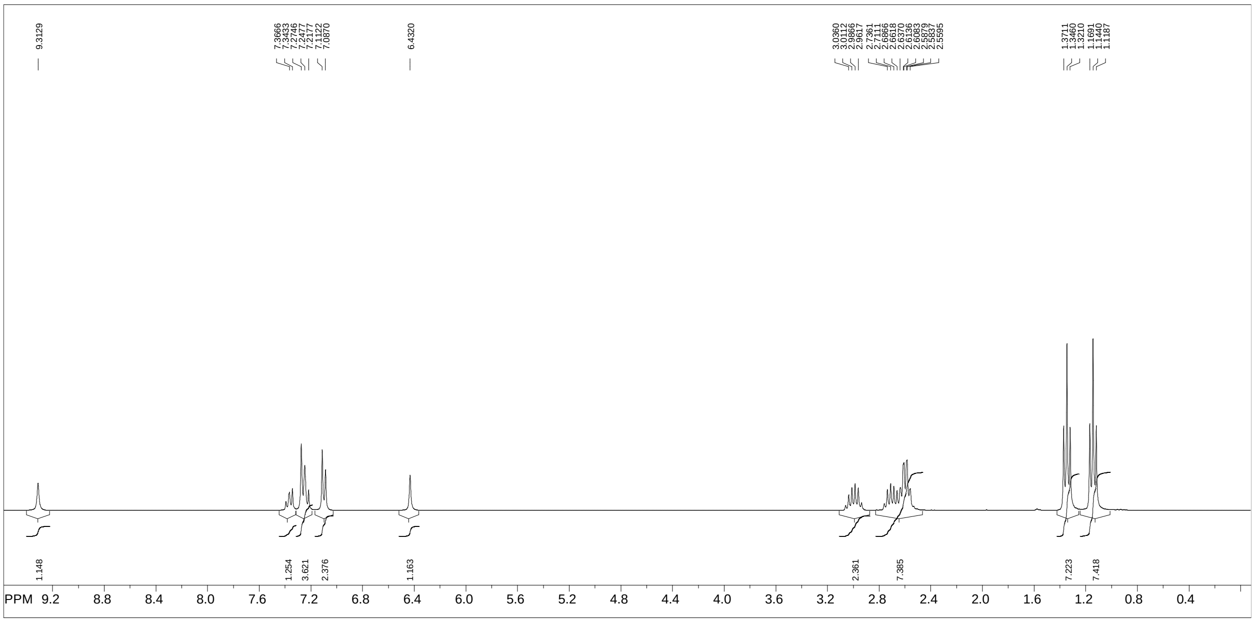

Figure S11. ^{1}H NMR spectrum of **2c**.

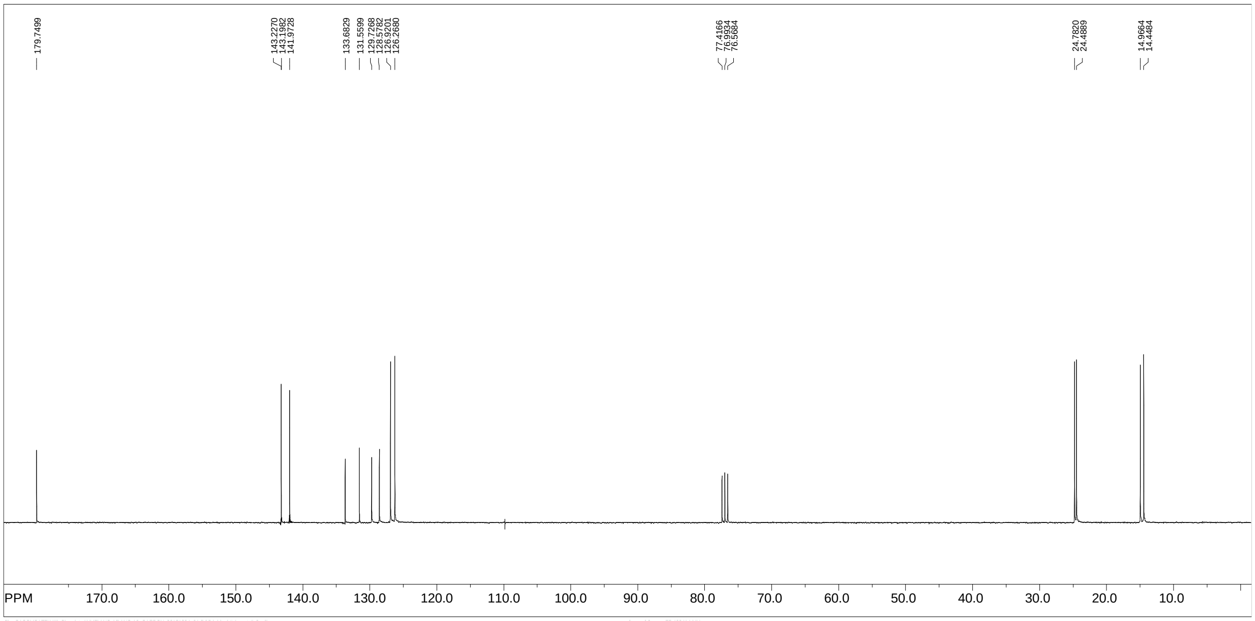

Figure S12. ^{13}C NMR spectrum of **2c**.
